# Multiple Reassorted Viruses as Cause of Highly Pathogenic Avian Influenza A(H5N8) Virus Epidemic, the Netherlands, 2016

**DOI:** 10.3201/eid2312.171062

**Published:** 2017-12

**Authors:** Nancy Beerens, Rene Heutink, Saskia A. Bergervoet, Frank Harders, Alex Bossers, Guus Koch

**Affiliations:** Wageningen Bioveterinary Research, Lelystad, the Netherlands

**Keywords:** highly pathogenic avian influenza virus, avian influenza virus, HPAIV, H5N8, complete genome sequencing, whole-genome sequencing, phylogenetic analysis, molecular clock, reassortment, influenza, viruses, outbreak, epidemic, the Netherlands, Holland, respiratory infections

## Abstract

In 2016, an epidemic of highly pathogenic avian influenza A virus subtype H5N8 in the Netherlands caused mass deaths among wild birds, and several commercial poultry farms and captive bird holdings were affected. We performed complete genome sequencing to study the relationship between the wild bird and poultry viruses. Phylogenetic analysis showed that the viruses are related to H5 clade 2.3.4.4 viruses detected in Russia in May 2016 but contained novel polymerase basic 2 and nucleoprotein gene segments and 2 different variants of the polymerase acidic segment. Molecular dating suggests that the reassortment events most likely occurred in wild birds in Russia or Mongolia. Furthermore, 2 genetically distinct H5N5 reassortant viruses were detected in wild birds in the Netherlands. Our study provides evidence for fast and continuing reassortment of H5 clade 2.3.4.4 viruses, which might lead to rapid changes in virus characteristics, such as pathogenicity, infectivity, transmission, and zoonotic potential.

Highly pathogenic avian influenza (HPAI) A virus subtype H5N8 was detected in wild birds found dead at Uvs-Nuur Lake at the border between Russia and Mongolia in May 2016. This discovery was considered an early warning for the potential spread of the virus by autumn migration of wild birds. On October 27, 2016, public health authorities in Hungary reported detection of HPAI H5N8 virus in a wild swan. Since then, H5N8 has been detected in many countries in Africa and Euriasia, including the Netherlands.

These reports constitute the fourth time intercontinental spread of an H5 HPAI virus has been observed. The gene segment encoding for the hemagglutinin (HA) surface protein of these viruses is a descendant of HPAI H5N1 virus (A/Goose/Guangdong/1/1996), first detected in China in 1996 (*1*). In wild birds, H5N1 virus was first reported in 2002 in Hong Kong and was most likely caused by spillover from infected poultry (*2*). In 2005, H5N1 virus was spread from Asia through Siberia to Europe, the Middle East, and Africa within a few months by migratory wild birds (*3*,*4*). In 2010, H5N1 virus was found in many countries in Asia and some in Europe (*5*). 

In 2014, HPAI H5N8 virus spread from Asia to Europe and North America (*6*,*7*), and 5 poultry holdings were infected in the Netherlands (*8*,*9*). In the Netherlands, the introduction of H5N8 in 2016 resulted in mass deaths among wild birds, and several commercial poultry holdings and captive birds were infected. The 2016 H5N8 virus appeared more pathogenic than the viruses in the earlier H5 HPAI epidemics, given that mass deaths of wild birds were never observed before. A recent study (*10*) showed that the H5N8 viruses identified in Russia in 2016 are reassortants, carrying 3 gene segments (HA, neuraminidase [NA], and nonstructural protein [NS]) originating from H5 clade 2.3.4.4 viruses circulating in eastern Asia. The other 5 gene segments (polymerase basic 1 and 2 [PB1 and PB2], polymerase acidic [PA], nucleoprotein [NP], and matrix protein [MP]) were found previously in low pathogenicity avian influenza (LPAI) viruses identified in Mongolia, China, and Vietnam.

In this study, we analyzed the genetic relationship between HPAI H5N8 viruses isolated from wild birds, commercial poultry, and captive birds in the Netherlands in 2016. Median-joining network analysis suggested that multiple separate introductions of the H5N8 virus occurred during the epidemic. Phylogenetic analysis showed that the virus evolved from the H5N8 viruses detected in Russia in 2016 by obtaining novel PB2, PA, and NP gene segments. Two different PA reassorted H5N8 viruses were introduced in the Netherlands, and we detected 2 distinct H5N5 viruses in wild birds. We performed molecular dating to estimate the timing of the reassortment events.

## Materials and Methods

### Virus Detection and Subtyping

We extracted influenza virus RNA from tracheal or cloacal swabs from dead wild birds by using MagNa Pure 96 (Roche, Basel, Switzerland). For commercial poultry farms, we used pools of 5 samples from clinically affected animals. We tested samples by using a matrix-gene real-time PCR, which detects all avian influenza virus subtypes, as described previously (*9*). We subtyped positive samples by using an H5-specific real-time PCR as recommended by the European Union reference laboratory (*11*). We determined the sequence of the HA cleavage site and the N subtype by using Sanger sequencing (*9*).

### Complete Genome Sequencing and Analysis

We purified influenza virus RNA by using the High Pure Viral RNA kit (Roche), amplified the RNA by using universal 8-segment primers, and then directly sequenced the RNA, as described previously (*12*). We sequenced purified amplicons at high coverage (average >1,000/nt position) using the Nextera XT DNA Library Preparation kit (Illumina, San Diego, CA, USA) and MiSeq paired-end 150-bp sequencing (Illumina). We mapped the reads by using the ViralProfiler-Workflow, an extension of the CLC Genomics Workbench (QIAGEN, Hilden, Germany). We generated consensus sequences by using a reference-based method. For generation of a defined set of influenza reference sequences, we clustered all complete genome sequences of Eurasia isolates in the GISAID EpiFlu database (https://www.gisaid.org) (years 2000–2016) per segment at 85% identity by using the CD-HIT-EST algorithm (*13*). We selected cluster representatives for each gene segment to generate a reference set. We first mapped reads to this reference set, and subsequently remapped them to the selected reference sequence. Finally, we extracted the consensus sequence of the complete virus genome. The ViralProfiler-Workflow also assigns the HA cleavage site and coding region of the proteins. We submitted sequences generated in this study to the GISAID database (Technical Appendix 1 Table 1).

### Phylogenetic Analysis

We performed phylogenetic analysis of the complete genome sequences for each genome segment separately. We generated alignments with the closest related viruses obtained from the GISAID database (Technical Appendix 1 Table 2) by using Muscle in MEGA6 (*14*). We performed molecular phylogenetic analysis by using the neighbor-joining method. We modeled the rate variation among sites with gamma distribution (shape parameter = 1) and used 1,000 bootstrap replicates to estimate branch support.

We manually concatenated the 8 gene segment alignments to generate a single alignment, which we used to construct phylogenetic networks by applying the median-joining method implemented in NETWORK as described (*15*). This model-free method uses a parsimony approach, based on pairwise differences, to connect each sequence to its closest neighbor. It allows creation of internal nodes (median vectors), which could be interpreted as unsampled or extinct ancestral genotypes to link the existing genotypes in the most parsimonious way.

### Molecular Dating

For each gene segment alignment, the simplest evolutionary model fitting the dataset was the Hasegaw–Kishino–Yano model with gammadistributed rates (*16*). We estimated nucleotide substitution rates by using Bayesian Markov chain Monte Carlo methods (*17*) until all parameters converged (chain lengths of 30 million delivered estimated sample sizes >500). We performed the analysis by using BEAST version 1.8.4 software (*18*) and strict or relaxed uncorrelated molecular clocks that we calibrated by using the sample isolation dates. We treated all gene segments separately to identify reassortants. We sampled the 30 million generations every 3,000 generations. We constructed a maximum clade credibility tree to summarize all 10,000 trees after removing the initial 10% burn-in by using TreeAnnotator (*17*). We visualized time-scaled phylogenetic trees by using FigTree version 1.4.2 (*19*) and further annotated the trees by using iTOL version 3.5.3 (*20*).

## Results

### Detection of HPAI H5N8 in Wild Birds and Poultry

**Figure 1:**
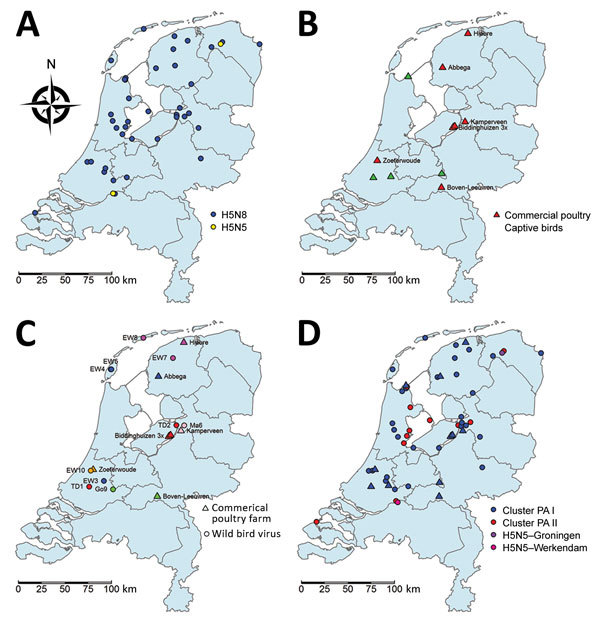
Geographic distribution of wild and captive birds and commercial poultry infected with highly pathogenic avian influenza A virus, the Netherlands, 2016. A) Location of dead wild birds infected with highly pathogenic avian influenza A virus subtypes H5N8 and H5N5; B) location of commercial poultry farms and captive birds infected with H5N8; C) location of H5N8-affected commercial poultry farms (open triangles), with the most identical wild bird viruses (open circles) shown in similar colors (wild bird virus isolates used for this analysis are numbered 1–10; Technical Appendix 1 Table 2); D) location of H5N8 viruses with gene segment PA I and PA II for commercial poultry farms and captive bird holdings and wild birds. Also shown are the H5N5 viruses isolated in Groningen and Werkendam. EW, Eurasian wigeon; Go, gray goose; Ma, mallard; PA, polymerase acidic; TD, tufted duck.

The 2016 H5N8 epidemic in the Netherlands started with occurrence of hundreds of dead tufted ducks (*Aythya fuligula*) around a lake near Monnickendam, in the central part of the country. These tufted ducks tested positive for H5N8 on November 10, 2016. Sequencing of the HA gene showed the polybasic amino acid sequence PLREKRRKR*GLF in the cleavage site, confirming its high pathogenicity. As the epidemic continued, wild birds around several lakes and water-rich areas in the central part of the country, and later also in the northern part, were infected. We plotted the geographic locations where the HPAI H5N8 virus was detected (Figure 1, panel A). Tufted ducks were the most affected bird species in November and Eurasian wigeons (*Anas penelope*) in December (*21*). However, many species of wild birds became infected, including diving and dabbling ducks as well as mute swans, grebes, gulls, and buzzards (*21*). An HPAI virus of subtype H5N5 was detected in 2 birds, a tufted duck found dead near Monnickendam, and a mute swan near Groningen.

The first introduction of H5N8 into a commercial poultry holding with Peking ducks was detected on November 25, in the municipality of Biddinghuizen. The farm was located in the central part of the country, a few kilometers from the lake where dead H5N8-positive wild birds were detected earlier. Severe clinical signs, such as lethargy, neurologic symptoms, and sudden death, were observed at the farm. Samples from the ducks tested positive by PCR, and the HPAI cleavage site (PLREKRRKR*GLF) was confirmed by sequencing. Several days later, samples from 2 other duck farms located within a distance of 3 km tested positive for H5N8. After these initial outbreaks, 5 other poultry holdings were affected in the central and northern parts of the country: 4 farms with laying hens, in Abbega, Boven-Leeuwen, Hiaure, and Zoeterwoude; and 1 duck farm, in Kamperveen. In addition, 4 introductions into captive birds were detected (1 black swan, 2 ducks, and 1 chicken). We noted the geographic locations of the infected commercial farms and captive bird holdings (Figure 1, panel B). All farms, with the exception of Boven-Leeuwen, were located in water-rich areas where dead H5N8-postive wild birds were found.

### Genetic Differences between Wild Bird and Poultry H5N8 Viruses

We performed deep sequencing to determine the complete genome sequence of 44 wild bird viruses, the viruses from 8 commercial poultry farms, and viruses from 4 captive birds in the Netherlands in 2016. We studied the genetic relationship between these viruses by median-joining network analysis, which showed that the viruses divide into 3 major clusters (Technical Appendix 1 Figure 1). Cluster A contained mainly Eurasian wigeons, whereas various wild bird species were found in clusters B and C (Technical Appendix 2 Figure 1). The viruses detected at the commercial poultry farms and in captive birds were located in clusters A and B, with the exception of those from Abbega. The network analysis showed no correlation between virus cluster and the geographic location of the virus. These results suggest that at least 3 separate introductions of the H5N8 virus occurred in the Netherlands, after which the virus likely spread to different parts of the country. In addition to the major clusters, we detected multiple separate viruses and small clusters, which suggests that more independent introductions of the H5N8 virus have occurred during the epidemic.

**Figure 2:**
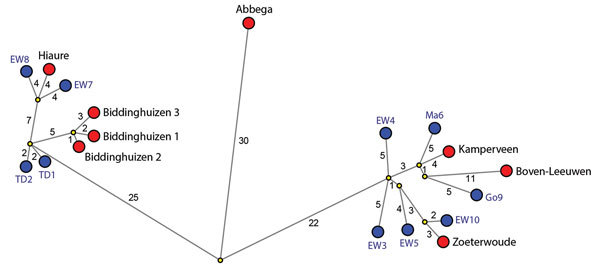
Median-joining network showing the genetic relationship between highly pathogenic avian influenza A viruses subtype H5N8 isolated from commercial poultry farms (red circles) and the most identical wild bird viruses (blue circles) found in the Netherlands, 2016. Predicted median vectors are shown in yellow. The length of the line represents the genetic distance, and the number of nucleotide changes is indicated. Wild bird virus isolates used for this analysis are numbered 1–10 (Technical Appendix 1 Table 2). GISAID EpiFlu database (https://www.gisaid.org) accession numbers are shown in Technical Appendix 1 Table 1. EW, Eurasian wigeon; Go, gray goose; Ma, mallard; TD,= tufted duck.

To analyze the genetic relationship between H5N8 viruses detected at commercial poultry farms and in wild birds, we identified the most identical wild bird virus for each farm (Technical Appendix 1 Table 2) and performed median-joining network analysis (Figure 2). The geographic locations of the poultry farms and the dead wild birds infected with the most related virus are shown (Figure 1, panel C). Epidemiologic investigation of the affected farms revealed no dangerous contacts. However, the 3 infected farms in Biddinghuizen were detected within a period of 7 days and are located within 3 km of one another. The median-joining network (Figure 2) showed that the complete genome sequence of the viruses from the farms in Biddinghuizen are highly similar, containing at most 5 nt changes. This fact indicates that the farms were either infected by direct transmission between farms or by separate introduction of virus from the same outside source. The viruses detected at the other 5 farms differed between 14 and 59 nt and are likely the result of separate introductions from wild birds (Figure 2). Closely related wild bird viruses with 5–9 nt changes were identified near the farms in Biddinghuizen, Hiaure, Kamperveen, and Zoeterwoude at 3–20 km distance. For the farm in Boven-Leeuwen, the closest related wildbird virus was found at a distance of 48 km and contained 16 nt differences. For the farm in Abbega, the most similar wild-bird virus contained 56 nt changes and was found at a distance of 52 km. We did not identify a more closely related virus, suggesting that the variation of wild bird viruses introduced into the Netherlands was larger than revealed in this study.

The comparison of wild bird and poultry sequences identified no specific mutations related to adaptation of the virus to poultry. Finally, the consensus sequence of the 2016 H5N8 virus was compared with the consensus sequence of the virus detected in the Netherlands in 2014, when 5 commercial poultry holdings were infected (results not shown). This analysis identified 97 nt differences, resulting in 18 aa changes in the HA gene segment between the consensus sequences of these viruses. In addition, we found 56 nt (14 aa) differences in the NA segment. In the 6 internal segments, we found a total of 142 aa differences in the open reading frames. The numerous genetic differences between the viruses indicate that the H5N8 virus found in the Netherlands in 2016 results from a new introduction and therefore is not from continuous circulation of the 2014 virus.

### Phylogenetic Analysis of the H5N8 Viruses

**Figure 3:**
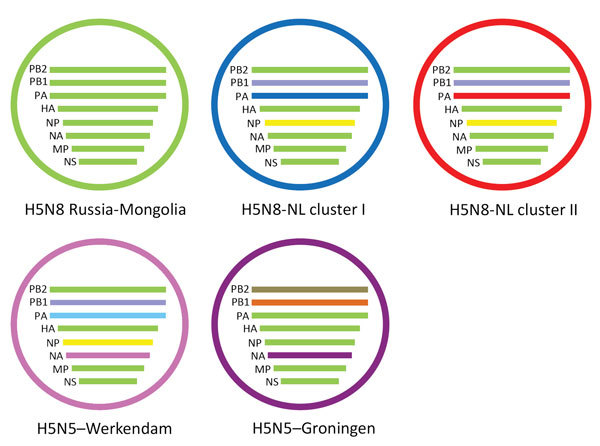
Schematic representation of the reassortant highly pathogenic avian influenza A virus subtypes H5N8 and H5N5 detected in the Netherlands, 2016. The Russia-Mongolia ancestor is shown in green. NL clusters 1 (blue) and 2 (red) viruses obtained novel PB2 and NP gene segments and 2 different PA segments. Two genetically distinct H5N5 viruses were detected, 1 in a tufted duck in Werkendam (pink) and 1 in a mute swan near Groningen (purple). HA, hemagglutinin; MP, matrix protein; NA, neuraminidase; NL, Netherlands; NP, nucleoprotein; NS, nonstructural protein; PA, polymerase acidic; PB1, polymerase basic 1; PB2, polymerase basic 2.

To study the origin of the H5N8 viruses found in the Netherlands in 2016, we performed a detailed phylogenetic analysis for all gene segments individually (Technical Appendix 2 Figure 2). This analysis confirms the differences between the 2014 and 2016 H5N8 viruses, which are found separate clusters. The closest relatives of the 2016 viruses in the Netherlands are the Russia–Mongolia H5N8 clade 2.3.4.4 viruses that were first detected in May 2016 in dead wild birds near Uvs-Nuur Lake. However, we identified several reassortments, which we have represented schematically (Figure 3). Five of the gene segments (PB 1, HA, NA, MP, and NS) are highly similar to the Russia–Mongolia viruses. However, the closest relatives of PB2 in the GISAID database are LPAI viruses found in Bangladesh and Russia (Technical Appendix 1 Table 1). The closest relatives of the NP gene segment are found in LPAI viruses in the Netherlands and the Republic of Georgia. An LPAI H7N9 virus detected in a commercial chicken holding in the Netherlands in June 2016 also carried a closely related NP segment. 

Phylogenetic analysis of the PA gene segment showed that the 2016 H5N8 viruses in the Netherlands divert into 2 separate clusters, designated PA I and PA II. Different subtypes of LPAI viruses found in Russia and Asia were identified as closest relatives for the 2 PA clusters (Technical Appendix 1 Table 3). Most of the viruses found in wild birds and all viruses detected in commercial poultry and captive birds are in cluster PA I. Cluster PA II contains 12 viruses isolated from various wild bird species and is represented by cluster C in the network analysis (Technical Appendix 2 Figure 1). These findings show that no correlation exists between PA cluster and bird species or geographic location (Technical Appendix 2 Figure 1 panels A and B; Figure 1, panel D). The viruses in the Netherlands evolved from the Russia–Mongolia H5N8 viruses by obtaining novel PB2 and NP segments and 2 variants of the PA segment. These results demonstrate that the H5N8 HPAI epidemic in the Netherlands is caused by 2 distinct reassorted viruses.

### Phylogenetic Analysis of Two H5N5 Viruses

Two HPAI H5N5 viruses were identified in dead wild birds in the Netherlands. For the tufted duck isolate (t_dk/NL-Werkendam/16014159–001/2016) PB1, PB2, HA, NP, MP, and NS are closely related to the H5N8 viruses detected in the Netherlands in 2016. The closest relatives of NA in the GISAID database are LPAI N5 viruses found in the Netherlands, Europe, and Asia in previous years (Technical Appendix 1 Table 3; Technical Appendix 2 Figure 1). For the PA segment, this H5N5 virus does not fall in either cluster PA I or PA II. The closest relatives of PA are LPAI viruses found in the Netherlands and Asia during 2012–2014. For the mute swan isolate (m-swan/NL-Groningen/16015825–001/2016), all genome segments are highly similar to an H5N5 virus identified in feces of a wild bird in Kamchatka, Russia, in October 2016. Phylogenetic analysis of the HA, PA, NP, MP, and NS segments shows that this H5N5 virus clusters with the Russia–Mongolia H5N8 viruses and not with the H5N8 2016 viruses in the Netherlands. The closest relatives of NA in the GISAID database are LPAI N5 viruses found in Asia in previous years. A similar N5 segment was also found in H5N5 viruses detected in Croatia, Italy, and Poland during the H5N8 epidemic in 2016–2017. The PB2 and PB1 segments cluster with various LPAI viruses detected earlier in Russia, Mongolia, and Europe. The mute swan virus thus is a direct descendant of a reassorted H5N5 virus from Russia, given that it was also previously detected in Kamchatka. We have depicted the genetic constitution of the 2 H5N5 viruses (Figure 3). These results indicate that 2 genetically distinct H5N5 viruses have been found in the Netherlands.

### Molecular Dating of H5N8 Reassortment Events

**Figure 4:**
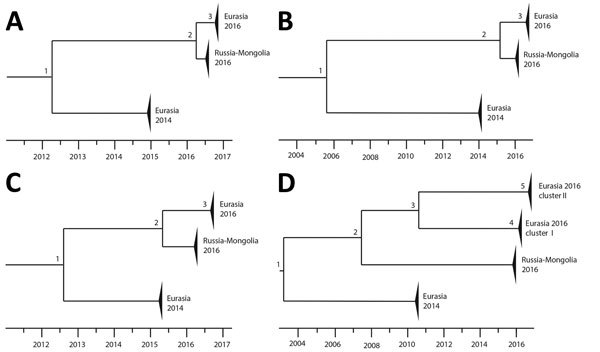
Schematic representation of the molecular dating analysis of highly pathogenic avian influenza A virus subtypes H5N8 and H5N5 detected in the Netherlands, 2016. Time-scaled phylogenetic trees are shown for 4 gene segments: A) hemagglutinin; B) polymerase basic 2; C) nucleoprotein; and D) polymerase acidic. For each of the numbered nodes, calculated time of most recent common ancestor, 95% highest posterior density interval, and posterior are listed in the Table.

To estimate the timing of the reassortment events that led to the emergence of the H5N8 viruses in the Netherlands, we performed molecular dating (Technical Appendix 2 Figure 3). We estimated the time of most recent common ancestor for all gene segments by using a relaxed molecular clock (Table). A schematic representation of the time-scaled phylogenetic tree for the HA, PB2, NP, and PA segments is shown (Figure 4). The H5N8 viruses in 2016 diverted from the 2014 viruses in April 2012 (Figure 4, panel A [node 1]), from the Russia–Mongolia H5N8 viruses in March 2016 (node 2), and we estimated the common ancestor of the viruses in the Netherlands to have occurred in August 2016 (node 3). Similar results were obtained for the PB 1, NA, MP, and NS gene segments (Table; Technical Appendix 2 Figure 3). The PB2 segment was transferred from LPAI viruses in June 2016 (Figure 4, panel B [node 3]) and the NP segment in August 2016 (Figure 4, panel C [node 3]). Two different PA segments were detected in H5N8 viruses in the Netherlands. The viruses in cluster PA I obtained the PA segment from LPAI viruses in April 2016 (Figure 4, panel D [node 4]) and in cluster PA II in August 2016 (Figure 4, panel D [node 5]). We also performed calculations by using a strict molecular clock, with similar outcomes (results not shown). Thus, the H5N8 viruses in the Netherlands were generated by multiple reassortment events with LPAI viruses, a process that was likely completed in August 2016. Because the H5N8 virus was introduced in the Netherlands in November 2016, this analysis suggests that the reassortment events took place in wild birds in Russia-Mongolia. Molecular dating for H5N5 shows that the common ancestor of the mute swan isolate and the Kamchatka isolate already circulated in January 2015. The N5 gene segments of the tufted duck and mute swan isolate diverted in 1989 (node A). Multiple reassorted viruses thus were introduced in the Netherlands by the autumn migration of wild birds from their breeding grounds in Russia-Mongolia.

**Table:** tMRCA of 8 gene segments of highly pathogenic avian influenza A virus subtype H5N8 in the Netherlands, determined by using a relaxed clock, with 95% HPD and posterior values*

Gene segment and node†	tMRCA	95% HPD interval		Posterior
		Begin	End	
Polymerase basic 2				
1	2005 Jul 21	1999 Apr 21	2011 Aug 2	1.00000
2	2015 Apr 13	2014 Aug 19	2015 Dec 23	1.00000
3	2016 Jul 2	2016 Apr 29	2016 Sep 6	1.00000
Polymerase basic 1				
1	2009 Feb 23	2006 Jun 27	2011 Feb 16	1.00000
2	2015 Dec 15	2015 Sep 12	2016 Mar 11	1.00000
3	2016 Aug 18	2016 Jul 3	2016 Sep 29	0.99610
Polymerase acidic				
1	2003 Jan 23	1998 Jun 20	2006 Nov 20	1.00000
2	2007 May 19	2005 Mar 31	2009 Jun 15	0.99070
3	2010 Jul 6	2009 Jul 20	2011 May 2	1.00000
4	2016 Apr 26	2016 Jan 10	2016 Jul 26	1.00000
5	2016 Aug 25	2016 Jun 23	2016 Oct 17	1.00000
Hemagglutinin				
1	2012 Apr 6	2010 Nov 14	2013 Jul 4	1.00000
2	2016 Mar 11	2016 Jan 15	2016 Apr 30	0.99420
3	2016 Aug 23	2016 Apr 8	2016 Aug 15	0.99989
Nucleoprotein				
1	2012 Jun 6	2010 Aug 16	2013 Aug 30	1.00000
2	2015 Apr 15	2014 Sep 11	2015 Dec 5	1.00000
3	2016 Aug 20	2016 Jun 28	2016 Oct 12	1.00000
Neuraminidase (N8)				
1	2011 Sep 5	2002 Nov 5	2013 Dec 24	1.00000
2	2016 Apr 14	2016 Mar 3	2016 May 14	0.91980
3	2016 Aug 31	2016 Jul 18	2016 Oct 8	0.76560
Matrix protein				
1	2011 Aug 29	2008 Sep 21	2013 Aug 21	1.00000
2	2015 Dec 9	2015 Jul 30	2016 Mar 16	1.00000
3	2016 Sep 9	2016 Jul 25	2016 Oct 16	0.97360
Nonstructural protein				
1	2010 May 17	2007 Jan 4	2012 Dec 7	1.00000
2	2015 Dec 9	2015 Jul 30	2016 Mar 18	1.00000
3	2016 Aug 23	2016 Jul 6	2016 Oct 2	0.99630
Neuraminidase (N5)				
A	1989 May 28	1983 Apr 24	1994 Apr 30	0.43740
B	2014 Apr 1	2012 Nov 27	2015 Apr 5	1.00000
C	2015 Jan 4	2013 Aug 11	2016 Jan 18	1.00000
D	2005 Sep 15	2004 Feb 13	2006 Nov 28	1.00000

*HPD, highest posterior density; tMRCA, calculated time of most recent common ancestor. †Nodes of the time-scaled phylogenetic tree are shown in Figure 4 and Technical Appendix 2 Figure 3.

## Discussion

The introduction of the HPAI H5N8 virus in the Netherlands in 2016 resulted in the death of many wild birds, and several commercial poultry and captive bird holdings were affected. We performed genetic analysis to study the relationship between wild bird and poultry viruses. Median-joining network analysis suggested that multiple separate introductions of the H5N8 virus occurred in the Netherlands. This analysis also demonstrated that the viruses on 5 infected farms were not closely related (Figure 2). On the 3 farms in Biddinghuizen, highly similar viruses were identified, which might have resulted from either farm-to-farm spread or separate introductions from the same source. For most poultry farms, a dead wild bird infected with a related virus was found near the farm. We observed numerous genetic differences between the H5N8 viruses introduced in the Netherlands in 2016 versus 2014, indicating the 2016 epidemic was caused by a new introduction and not by continuous circulation of the 2014 virus.

Phylogenetic analysis showed that the H5N8 viruses introduced in the Netherlands in 2016 are novel reassortants of the Russia–Mongolia H5 clade 2.3.4.4 viruses. The virus obtained new PB2 and NP segments, and we detected 2 different PA segments in H5N8 viruses in the Netherlands. We found no correlation between PA segment and bird species between PA segment and geographic location (Technical Appendix 2 Figure 1 [cluster C]). Molecular dating suggests that these reassortment events were completed in August 2016 and thus most likely occurred in wild birds in Russia-Mongolia. A recent genetic analysis of 2 wild bird and several poultry H5N8 viruses in Germany also identified the NP and PA reassortments (*22*). In addition, we identified PB2 as a reassortment on the basis of molecular dating studies and its similarity to PB2 in LPAI viruses. The PA II gene segment was not observed in H5N8 viruses in Germany or other countries in Europe, based on the analysis of sequences that are currently available. Furthermore, 2 genetically distinct H5N5 viruses have been found in the Netherlands. An analysis of the outbreak in Italy (*23*), published after submission of this manuscript, also showed multiple independent introductions of H5N8 and H5N5 viruses. Those findings provide evidence for rapid and continuing reassortment of the H5 clade 2.3.4.4 viruses, which allow the virus to change its genetic architecture very quickly and might increase the ability of the virus to infect poultry or humans in the future. Hence, extensive surveillance of wild bird populations in the border area of Russia and Mongolia and in common breeding grounds in northern Siberia appears essential to enable early warning of novel reassortants and sequence mutations of H5 clade 2.3.4.4 viruses.

Technical Appendix 1 Virus isolates and genetic analysis of an outbreak of highly pathogenic avian influenza A virus subtype H5N8, Netherlands, 2016.

Technical Appendix 2 Phylogenetic analyses of an outbreak of highly pathogenic avian influenza A virus subtype H5N8, Netherlands, 2016.
