# Supplementary material for: Multiple Reassorted Viruses as Cause of Highly Pathogenic Avian Influenza A(H5N8) Virus Epidemic, the Netherlands, 2016
Source: Emerg Infect Dis. 2017 Dec;23(12):1974–81. doi: 10.3201/eid2312.171062 (PMC5708218; doi:10.3201/eid2312.171062)
Supplement: Technical Appendix 2 — Phylogenetic analyses of an outbreak of highly pathogenic avian influenza A virus subtype H5N8, Netherlands, 2016. [file 17-1062-Techapp-s2.pdf]

Article DOI: https://doi.org/10.3201/eid2312.171062

Multiple Reassorted Viruses as Cause of Highly Pathogenic Avian Influenza A(H5N8) Virus Epidemic, the Netherlands, 2016

Technical Appendix 2

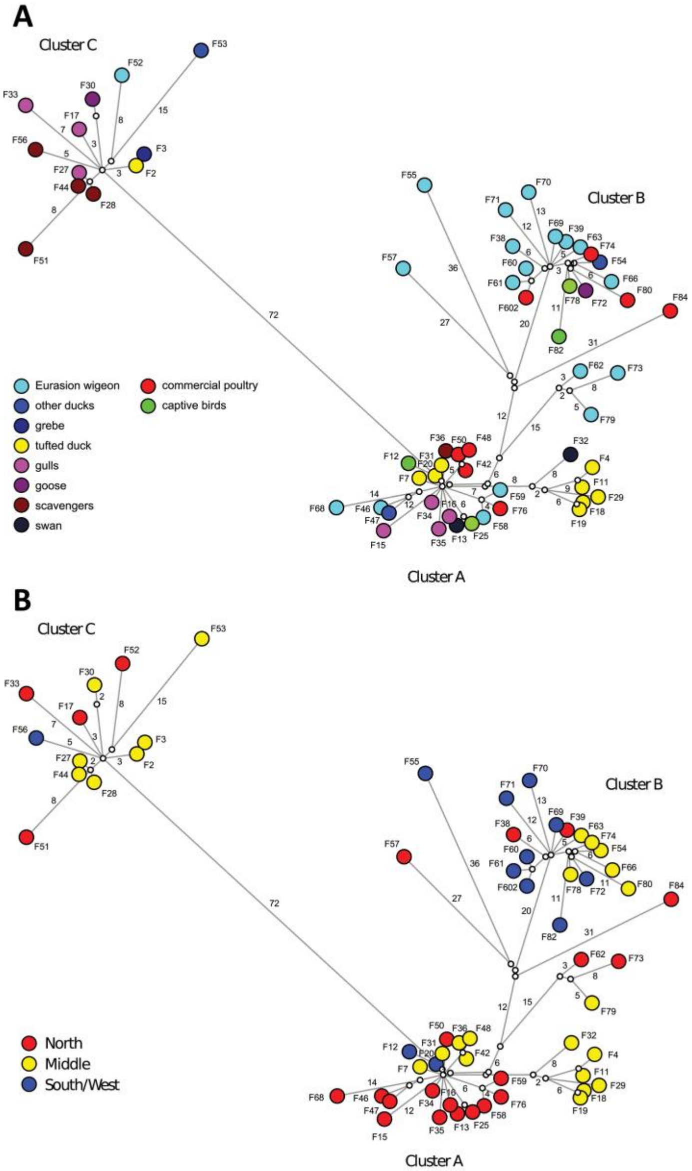

Technical Appendix 2 Figure 1. Network analysis: Median joining network of concatenated 8 gene segments. The network included all the most parsimonious trees linking the sequences. The virus isolates

are represented by circles, and their numbering is listed (Technical Appendix 1 Table 3). The smaller red circles are the intermediate vectors. Numbers (red) refer to the number of mutations between genotypes and/or median vectors. The GISAID accession numbers of the virus isolates (F-numbers) are listed in online Technical Appendix 1 Table 1.

Technical Appendix 2 Figure 2. Phylogenetic trees: The evolutionary history was inferred using the Neighbor-Joining method (*1*). The optimal trees for each gene segment are shown. One sequence for every virus isolated from a wild bird, poultry or captive bird is shown. The percentage of replicate trees in which the associated taxa clustered together in the bootstrap test (1,000 replicates) are shown next to the branches The tree is drawn to scale, with branch lengths in the same units as those of the evolutionary distances used to infer the phylogenetic tree. The evolutionary distances were computed using the Tamura-Nei method (*2*) and are in the units of the number of base substitutions per site. The rate variation among sites was modeled with a gamma distribution (shape parameter = 1). Evolutionary analyses were conducted in MEGA6 (*3*). The GISAID accession numbers of the viruses used in this study are listed in online Technical Appendix 1 Tables 1 and 2. The H5N8 viruses isolated in Russia-Mongolia are marked in green, the H5N8 viruses isolated in the Netherlands in 2016 and harboring the PA I gene segment are marked in bleu, those having the PA II gene segment in red. The H5N5 virus isolated from a tufted duck near Werkendam is marked in pink, that isolated from a mute swan near Groningen in purple.

A

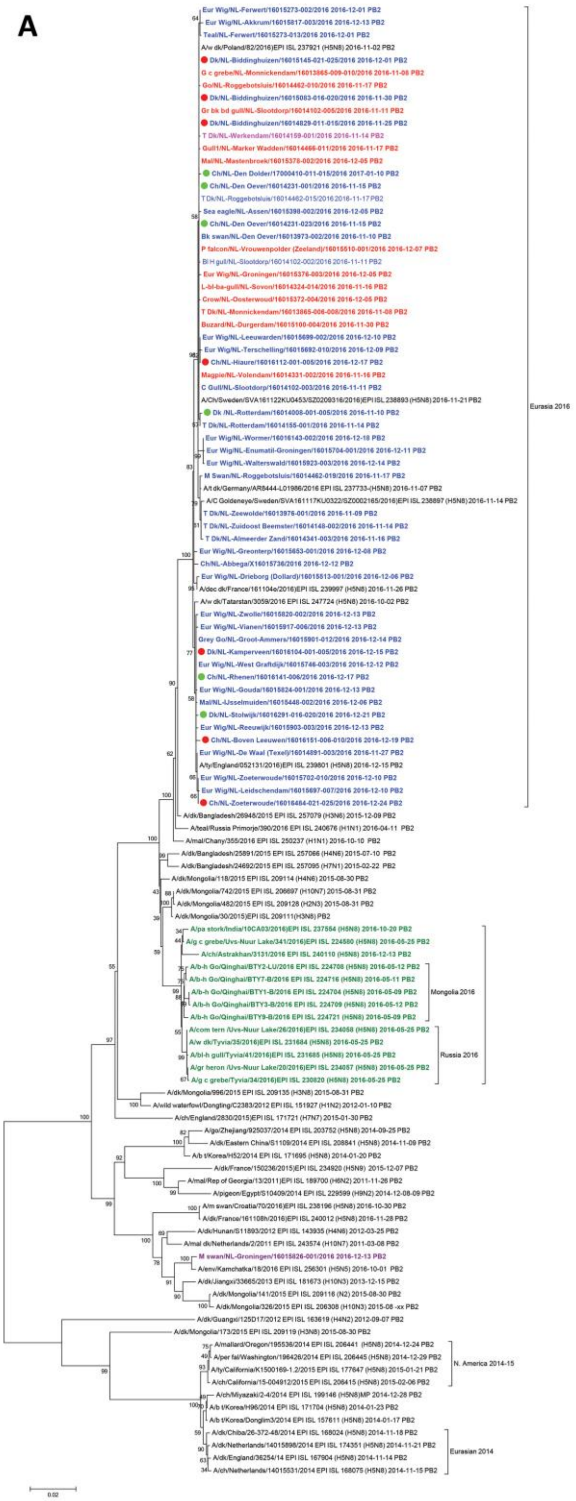
Eurasia 2016
Mongolia 2016
Russia 2016
N. America 2014-15
Eurasian 2014
0.02

B

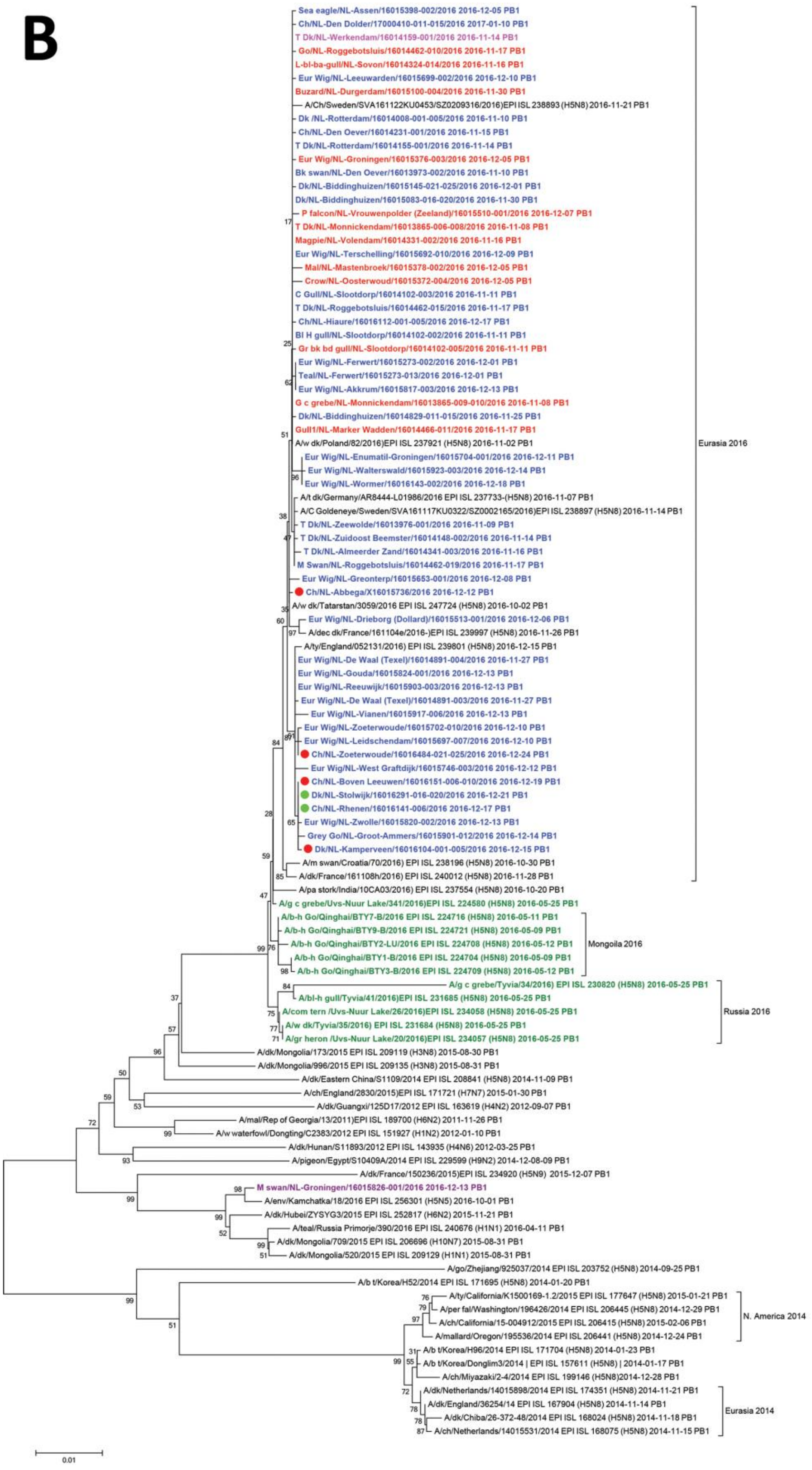

Sea eagle/NL-Assen/16015398-002/2016 2016-12-05 PB1
Ch/NL-Den Dolder/17000410-011-015/2016 2017-01-10 PB1
T Dk/NL-Werkendam/16014159-001/2016 2016-11-14 PB1
Go/NL-Roggebotsluis/16014462-010/2016 2016-11-17 PB1
L-bl-ba-gull/NL-Sovon/16014324-014/2016 2016-11-16 PB1
Eur Wig/NL-Leeuwarden/16015699-002/2016 2016-12-10 PB1
Buzard/NL-Durgerdam/16015100-004/2016 2016-11-30 PB1
A/Ch/Sweden/SVA161122KU0453/SZ0209316/2016)EPI ISL 238893 (H5N8) 2016-11-21 PB1
Dk /NL-Rotterdam/16014008-001-005/2016 2016-11-10 PB1
Ch/NL-Den Oever/16014231-001/2016 2016-11-15 PB1
T Dk/NL-Rotterdam/16014155-001/2016 2016-11-14 PB1
Eur Wig/NL-Groningen/16015376-003/2016 2016-12-05 PB1
Bk swan/NL-Den Oever/16013973-002/2016 2016-11-10 PB1
Dk/NL-Biddinghuizen/16015145-021-025/2016 2016-12-01 PB1
Dk/NL-Biddinghuizen/16015083-016-020/2016 2016-11-30 PB1
P falcon/NL-Vrouwenpolder (Zeeland)/16015510-001/2016 2016-12-07 PB1
T Dk/NL-Monnickendam/16013865-006-008/2016 2016-11-08 PB1
Magpie/NL-Volendam/16014331-002/2016 2016-11-16 PB1
Eur Wig/NL-Terschelling/16015692-010/2016 2016-12-09 PB1
Mal/NL-Mastenbroek/16015378-002/2016 2016-12-05 PB1
Crow/NL-Oosterwoud/16015372-004/2016 2016-12-05 PB1
C Gull/NL-Slootdorp/16014102-003/2016 2016-11-11 PB1
T Dk/NL-Roggebotsluis/16014462-015/2016 2016-11-17 PB1
Ch/NL-Hiaure/16016112-001-005/2016 2016-12-17 PB1
Bl H gull/NL-Slootdorp/16014102-002/2016 2016-11-11 PB1
Gr bk bd gull/NL-Slootdorp/16014102-005/2016 2016-11-11 PB1
Eur Wig/NL-Ferwert/16015273-002/2016 2016-12-01 PB1
Teal/NL-Ferwert/16015273-013/2016 2016-12-01 PB1
Eur Wig/NL-Akkrum/16015817-003/2016 2016-12-13 PB1
G c grebe/NL-Monnickendam/16013865-009-010/2016 2016-11-08 PB1
Dk/NL-Biddinghuizen/16014829-011-015/2016 2016-11-25 PB1
Gull1/NL-Marker Wadden/16014466-011/2016 2016-11-17 PB1
A/w dk/Poland/82/2016)EPI ISL 237921 (H5N8) 2016-11-02 PB1
Eur Wig/NL-Enumatil-Groningen/16015704-001/2016 2016-12-11 PB1
Eur Wig/NL-Walterswald/16015923-003/2016 2016-12-14 PB1
Eur Wig/NL-Wormer/16016143-002/2016 2016-12-18 PB1
A/t dk/Germany/AR8444-L01986/2016 EPI ISL 237733-(H5N8) 2016-11-07 PB1
A/C Goldeneye/Sweden/SVA161117KU0322/SZ0002165/2016)EPI ISL 238897 (H5N8) 2016-11-14 PB1
T Dk/NL-Zeewolde/16013976-001/2016 2016-11-09 PB1
T Dk/NL-Zuidoost Beemster/16014148-002/2016 2016-11-14 PB1
T Dk/NL-Almeerder Zand/16014341-003/2016 2016-11-16 PB1
M Swan/NL-Roggebotsluis/16014462-019/2016 2016-11-17 PB1
Eur Wig/NL-Greonterp/16015653-001/2016 2016-12-08 PB1
Ch/NL-Abbega/X16015736/2016 2016-12-12 PB1
A/w dk/Tatarstan/3059/2016 EPI ISL 247724 (H5N8) 2016-10-02 PB1
Eur Wig/NL-Drieborg (Dollard)/16015513-001/2016 2016-12-06 PB1
A/dec dk/France/161104e/2016-)EPI ISL 239997 (H5N8) 2016-11-26 PB1
A/ty/England/052131/2016) EPI ISL 239801 (H5N8) 2016-12-15 PB1
Eur Wig/NL-De Waal (Texel)/16014891-004/2016 2016-11-27 PB1
Eur Wig/NL-Gouda/16015824-001/2016 2016-12-13 PB1
Eur Wig/NL-Reeuwijk/16015903-003/2016 2016-12-13 PB1
Eur Wig/NL-De Waal (Texel)/16014891-003/2016 2016-11-27 PB1
Eur Wig/NL-Vianen/16015917-006/2016 2016-12-13 PB1
Eur Wig/NL-Zoeterwoude/16015702-010/2016 2016-12-10 PB1
Eur Wig/NL-Leidschendam/16015697-007/2016 2016-12-10 PB1
Ch/NL-Zoeterwoude/16016484-021-025/2016 2016-12-24 PB1
Eur Wig/NL-West Graftdijk/16015746-003/2016 2016-12-12 PB1
Ch/NL-Boven Leeuwen/16016151-006-010/2016 2016-12-19 PB1
Dk/NL-Stolwijk/16016291-016-020/2016 2016-12-21 PB1
Ch/NL-Rhenen/16016141-006/2016 2016-12-17 PB1
Eur Wig/NL-Zwolle/16015820-002/2016 2016-12-13 PB1
Grey Go/NL-Groot-Ammers/16015901-012/2016 2016-12-14 PB1
Dk/NL-Kamperveen/16016104-001-005/2016 2016-12-15 PB1
A/m swan/Croatia/70/2016) EPI ISL 238196 (H5N8) 2016-10-30 PB1
A/dk/France/161108h/2016) EPI ISL 240012 (H5N8) 2016-11-28 PB1
Eurasia 2016
A/pa stork/India/10CA03/2016) EPI ISL 237554 (H5N8) 2016-10-20 PB1
A/g c grebe/Uvs-Nuur Lake/341/2016)EPI ISL 224580 (H5N8) 2016-05-25 PB1
A/b-h Go/Qinghai/BTY7-B/2016 EPI ISL 224716 (H5N8) 2016-05-11 PB1
A/b-h Go/Qinghai/BTY9-B/2016 EPI ISL 224721 (H5N8) 2016-05-09 PB1
A/b-h Go/Qinghai/BTY2-LU/2016 EPI ISL 224708 (H5N8) 2016-05-12 PB1
A/b-h Go/Qinghai/BTY1-B/2016 EPI ISL 224704 (H5N8) 2016-05-09 PB1
A/b-h Go/Qinghai/BTY3-B/2016 EPI ISL 224709 (H5N8) 2016-05-12 PB1
Mongoila 2016
A/g c grebe/Tyvia/34/2016) EPI ISL 230820 (H5N8) 2016-05-25 PB1
A/bl-h gull/Tyvia/41/2016)EPI ISL 231685 (H5N8) 2016-05-25 PB1
A/com tern /Uvs-Nuur Lake/26/2016)EPI ISL 234058 (H5N8) 2016-05-25 PB1
A/w dk/Tyvia/35/2016) EPI ISL 231684 (H5N8) 2016-05-25 PB1
A/gr heron /Uvs-Nuur Lake/20/2016)EPI ISL 234057 (H5N8) 2016-05-25 PB1
Russia 2016
A/dk/Mongolia/173/2015 EPI ISL 209119 (H3N8) 2015-08-30 PB1
A/dk/Mongolia/996/2015 EPI ISL 209135 (H3N8) 2015-08-31 PB1
A/dk/Eastern China/S1109/2014 EPI ISL 208841 (H5N8) 2014-11-09 PB1
A/ch/England/2830/2015)EPI ISL 171721 (H7N7) 2015-01-30 PB1
A/dk/Guangxi/125D17/2012 EPI ISL 163619 (H4N2) 2012-09-07 PB1
A/mal/Rep of Georgia/13/2011)EPI ISL 189700 (H6N2) 2011-11-26 PB1
A/w waterfowl/Dongting/C2383/2012 EPI ISL 151927 (H1N2) 2012-01-10 PB1
A/dk/Hunan/S11893/2012 EPI ISL 143935 (H4N6) 2012-03-25 PB1
A/pigeon/Egypt/S10409A/2014 EPI ISL 229599 (H9N2) 2014-12-08-09 PB1
A/dk/France/150236/2015)EPI ISL 234920 (H5N9) 2015-12-07 PB1
M swan/NL-Groningen/16015826-001/2016 2016-12-13 PB1
A/env/Kamchatka/18/2016 EPI ISL 256301 (H5N5) 2016-10-01 PB1
A/dk/Hubei/ZYSYG3/2015 EPI ISL 252817 (H6N2) 2015-11-21 PB1
A/teal/Russia Primorje/390/2016 EPI ISL 240676 (H1N1) 2016-04-11 PB1
A/dk/Mongolia/709/2015 EPI ISL 206696 (H10N7) 2015-08-31 PB1
A/dk/Mongolia/520/2015 EPI ISL 209129 (H1N1) 2015-08-31 PB1
A/go/Zhejiang/925037/2014 EPI ISL 203752 (H5N8) 2014-09-25 PB1
A/b t/Korea/H52/2014 EPI ISL 171695 (H5N8) 2014-01-20 PB1
A/ty/California/K1500169-1.2/2015 EPI ISL 177647 (H5N8) 2015-01-21 PB1
A/per fal/Washington/196426/2014 EPI ISL 206445 (H5N8) 2014-12-29 PB1
A/ch/California/15-004912/2015 EPI ISL 206415 (H5N8) 2015-02-06 PB1
A/mallard/Oregon/195536/2014 EPI ISL 206441 (H5N8) 2014-12-24 PB1
N. America 2014
A/b t/Korea/H96/2014 EPI ISL 171704 (H5N8) 2014-01-23 PB1
A/b t/Korea/Donglim3/2014 | EPI ISL 157611 (H5N8) | 2014-01-17 PB1
A/ch/Miyazaki/2-4/2014 EPI ISL 199146 (H5N8)2014-12-28 PB1
A/dk/Netherlands/14015898/2014 EPI ISL 174351 (H5N8) 2014-11-21 PB1
A/dk/England/36254/14 EPI ISL 167904 (H5N8) 2014-11-14 PB1
A/dk/Chiba/26-372-48/2014 EPI ISL 168024 (H5N8) 2014-11-18 PB1
A/ch/Netherlands/14015531/2014 EPI ISL 168075 (H5N8) 2014-11-15 PB1
Eurasia 2014
0.01

C

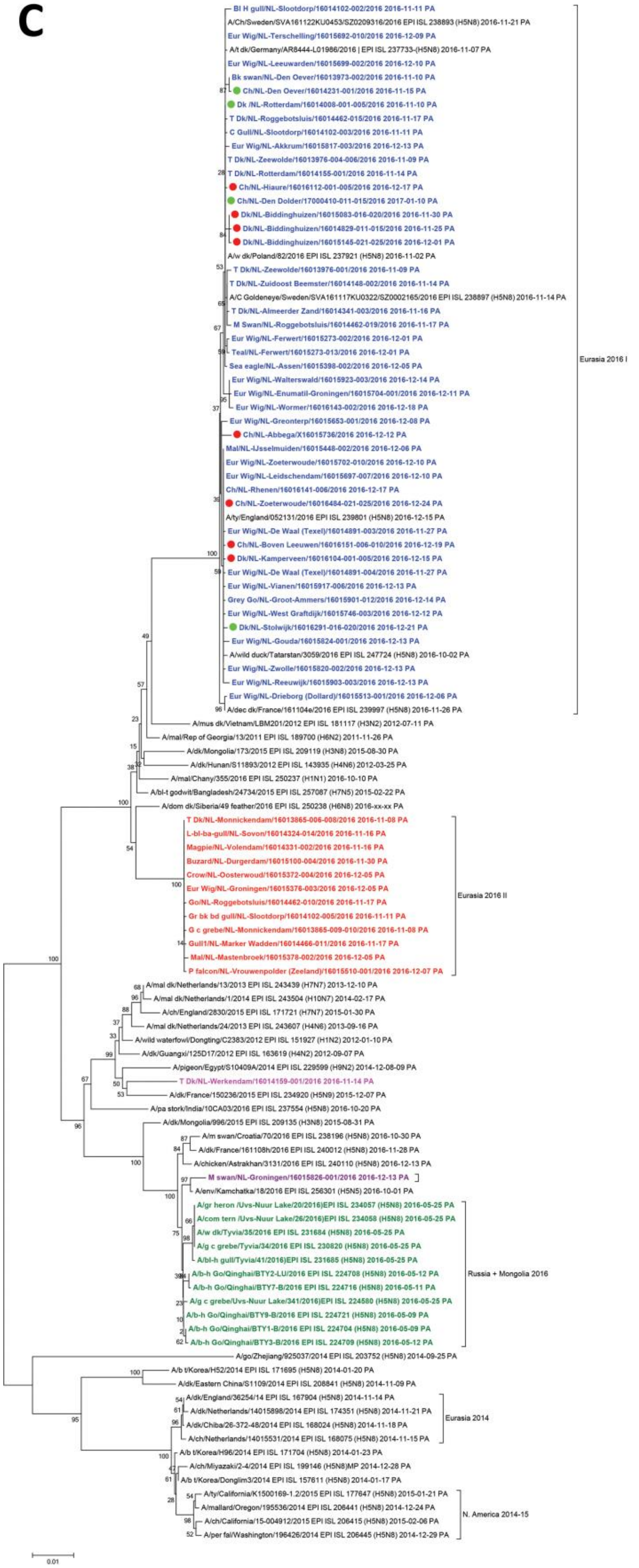
Eurasia 2016 I
Eurasia 2016 II
Russia + Mongolia 2016
Eurasia 2014
N. America 2014-15
0.01

D

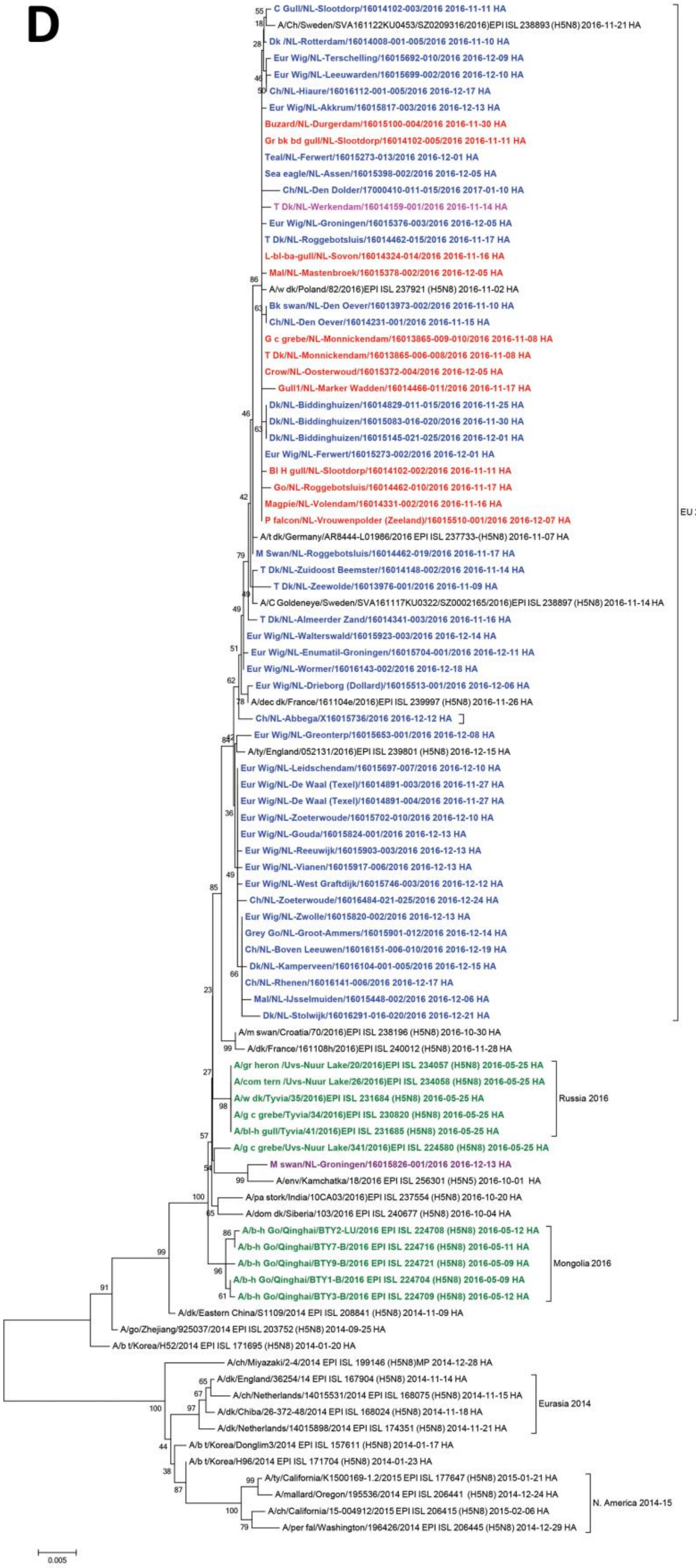

C Gull/NL-Slootdorp/16014102-003/2016 2016-11-11 HA
A/Ch/Sweden/SVA161122KU0453/SZ0209316/2016)EPI ISL 238893 (H5N8) 2016-11-21 HA
Dk /NL-Rotterdam/16014008-001-005/2016 2016-11-10 HA
Eur Wig/NL-Terschelling/16015692-010/2016 2016-12-09 HA
Eur Wig/NL-Leeuwarden/16015699-002/2016 2016-12-10 HA
Ch/NL-Hiaure/16016112-001-005/2016 2016-12-17 HA
Eur Wig/NL-Akkrum/16015817-003/2016 2016-12-13 HA
Buzard/NL-Durgerdam/16015100-004/2016 2016-11-30 HA
Gr bk bd gull/NL-Slootdorp/16014102-005/2016 2016-11-11 HA
Teal/NL-Ferwert/16015273-013/2016 2016-12-01 HA
Sea eagle/NL-Assen/16015398-002/2016 2016-12-05 HA
Ch/NL-Den Dolder/17000410-011-015/2016 2017-01-10 HA
T Dk/NL-Werkendam/16014159-001/2016 2016-11-14 HA
Eur Wig/NL-Groningen/16015376-003/2016 2016-12-05 HA
T Dk/NL-Roggebotsluis/16014462-015/2016 2016-11-17 HA
L-bl-ba-gull/NL-Sovon/16014324-014/2016 2016-11-16 HA
Mal/NL-Mastenbroek/16015378-002/2016 2016-12-05 HA
A/w dk/Poland/82/2016)EPI ISL 237921 (H5N8) 2016-11-02 HA
Bk swan/NL-Den Oever/16013973-002/2016 2016-11-10 HA
Ch/NL-Den Oever/16014231-001/2016 2016-11-15 HA
G c grebe/NL-Monnickendam/16013865-009-010/2016 2016-11-08 HA
T Dk/NL-Monnickendam/16013865-006-008/2016 2016-11-08 HA
Crow/NL-Oosterwoud/16015372-004/2016 2016-12-05 HA
Gull1/NL-Marker Wadden/16014466-011/2016 2016-11-17 HA
Dk/NL-Biddinghuizen/16014829-011-015/2016 2016-11-25 HA
Dk/NL-Biddinghuizen/16015083-016-020/2016 2016-11-30 HA
Dk/NL-Biddinghuizen/16015145-021-025/2016 2016-12-01 HA
Eur Wig/NL-Ferwert/16015273-002/2016 2016-12-01 HA
Bl H gull/NL-Slootdorp/16014102-002/2016 2016-11-11 HA
Go/NL-Roggebotsluis/16014462-010/2016 2016-11-17 HA
Magpie/NL-Volendam/16014331-002/2016 2016-11-16 HA
P falcon/NL-Vrouwenpolder (Zeeland)/16015510-001/2016 2016-12-07 HA
A/t dk/Germany/AR8444-L01986/2016 EPI ISL 237733-(H5N8) 2016-11-07 HA
M Swan/NL-Roggebotsluis/16014462-019/2016 2016-11-17 HA
T Dk/NL-Zuidoost Beemster/16014148-002/2016 2016-11-14 HA
T Dk/NL-Zeewolde/16013976-001/2016 2016-11-09 HA
A/C Goldeneye/Sweden/SVA161117KU0322/SZ0002165/2016)EPI ISL 238897 (H5N8) 2016-11-14 HA
T Dk/NL-Almeerder Zand/16014341-003/2016 2016-11-16 HA
Eur Wig/NL-Walterswald/16015923-003/2016 2016-12-14 HA
Eur Wig/NL-Enumatil-Groningen/16015704-001/2016 2016-12-11 HA
Eur Wig/NL-Wormer/16016143-002/2016 2016-12-18 HA
Eur Wig/NL-Drieborg (Dollard)/16015513-001/2016 2016-12-06 HA
A/dec dk/France/161104e/2016)EPI ISL 239997 (H5N8) 2016-11-26 HA
Ch/NL-Abbega/X16015736/2016 2016-12-12 HA
Eur Wig/NL-Greonterp/16015653-001/2016 2016-12-08 HA
A/ty/England/052131/2016)EPI ISL 239801 (H5N8) 2016-12-15 HA
Eur Wig/NL-Leidschendam/16015697-007/2016 2016-12-10 HA
Eur Wig/NL-De Waal (Texel)/16014891-003/2016 2016-11-27 HA
Eur Wig/NL-De Waal (Texel)/16014891-004/2016 2016-11-27 HA
Eur Wig/NL-Zoeterwoude/16015702-010/2016 2016-12-10 HA
Eur Wig/NL-Gouda/16015824-001/2016 2016-12-13 HA
Eur Wig/NL-Reeuwijk/16015903-003/2016 2016-12-13 HA
Eur Wig/NL-Vianen/16015917-006/2016 2016-12-13 HA
Eur Wig/NL-West Graftdijk/16015746-003/2016 2016-12-12 HA
Ch/NL-Zoeterwoude/16016484-021-025/2016 2016-12-24 HA
Eur Wig/NL-Zwolle/16015820-002/2016 2016-12-13 HA
Grey Go/NL-Groot-Ammers/16015901-012/2016 2016-12-14 HA
Ch/NL-Boven Leeuwen/16016151-006-010/2016 2016-12-19 HA
Dk/NL-Kamperveen/16016104-001-005/2016 2016-12-15 HA
Ch/NL-Rhenen/16016141-006/2016 2016-12-17 HA
Mal/NL-IJsselmuiden/16015448-002/2016 2016-12-06 HA
Dk/NL-Stolwijk/16016291-016-020/2016 2016-12-21 HA
EU 2016
A/m swan/Croatia/70/2016)EPI ISL 238196 (H5N8) 2016-10-30 HA
A/dk/France/161108h/2016)EPI ISL 240012 (H5N8) 2016-11-28 HA
A/gr heron /Uvs-Nuur Lake/20/2016)EPI ISL 234057 (H5N8) 2016-05-25 HA
A/com tern /Uvs-Nuur Lake/26/2016)EPI ISL 234058 (H5N8) 2016-05-25 HA
A/w dk/Tyvia/35/2016)EPI ISL 231684 (H5N8) 2016-05-25 HA
A/g c grebe/Tyvia/34/2016)EPI ISL 230820 (H5N8) 2016-05-25 HA
A/bl-h gull/Tyvia/41/2016)EPI ISL 231685 (H5N8) 2016-05-25 HA
Russia 2016
A/g c grebe/Uvs-Nuur Lake/341/2016)EPI ISL 224580 (H5N8) 2016-05-25 HA
M swan/NL-Groningen/16015826-001/2016 2016-12-13 HA
A/env/Kamchatka/18/2016 EPI ISL 256301 (H5N5) 2016-10-01 HA
A/pa stork/India/10CA03/2016)EPI ISL 237554 (H5N8) 2016-10-20 HA
A/dom dk/Siberia/103/2016 EPI ISL 240677 (H5N8) 2016-10-04 HA
A/b-h Go/Qinghai/BTY2-LU/2016 EPI ISL 224708 (H5N8) 2016-05-12 HA
A/b-h Go/Qinghai/BTY7-B/2016 EPI ISL 224716 (H5N8) 2016-05-11 HA
A/b-h Go/Qinghai/BTY9-B/2016 EPI ISL 224721 (H5N8) 2016-05-09 HA
A/b-h Go/Qinghai/BTY1-B/2016 EPI ISL 224704 (H5N8) 2016-05-09 HA
A/b-h Go/Qinghai/BTY3-B/2016 EPI ISL 224709 (H5N8) 2016-05-12 HA
Mongolia 2016
A/dk/Eastern China/S1109/2014 EPI ISL 208841 (H5N8) 2014-11-09 HA
A/go/Zhejiang/925037/2014 EPI ISL 203752 (H5N8) 2014-09-25 HA
A/b t/Korea/H52/2014 EPI ISL 171695 (H5N8) 2014-01-20 HA
A/ch/Miyazaki/2-4/2014 EPI ISL 199146 (H5N8)MP 2014-12-28 HA
A/dk/England/36254/14 EPI ISL 167904 (H5N8) 2014-11-14 HA
A/ch/Netherlands/14015531/2014 EPI ISL 168075 (H5N8) 2014-11-15 HA
A/dk/Chiba/26-372-48/2014 EPI ISL 168024 (H5N8) 2014-11-18 HA
A/dk/Netherlands/14015898/2014 EPI ISL 174351 (H5N8) 2014-11-21 HA
Eurasia 2014
A/b t/Korea/Donglim3/2014 EPI ISL 157611 (H5N8) 2014-01-17 HA
A/b t/Korea/H96/2014 EPI ISL 171704 (H5N8) 2014-01-23 HA
A/ty/California/K1500169-1.2/2015 EPI ISL 177647 (H5N8) 2015-01-21 HA
A/mallard/Oregon/195536/2014 EPI ISL 206441 (H5N8) 2014-12-24 HA
A/ch/California/15-004912/2015 EPI ISL 206415 (H5N8) 2015-02-06 HA
A/per fal/Washington/196426/2014 EPI ISL 206445 (H5N8) 2014-12-29 HA
N. America 2014-15
0.005

E

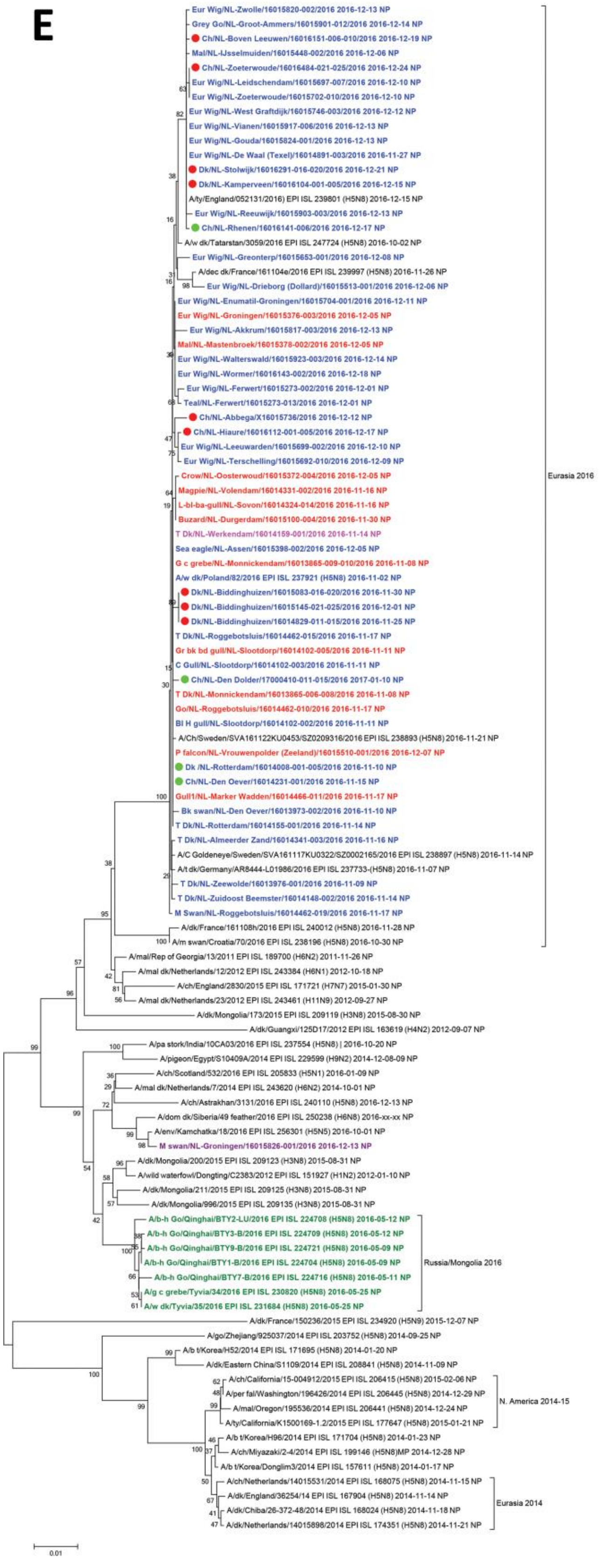

Eur Wig/NL-Zwolle/16015820-002/2016 2016-12-13 NP
Grey Go/NL-Groot-Ammers/16015901-012/2016 2016-12-14 NP
Ch/NL-Boven Leeuwen/16016151-006-010/2016 2016-12-19 NP
Mal/NL-IJsselmuiden/16015448-002/2016 2016-12-06 NP
Ch/NL-Zoeterwoude/16016484-021-025/2016 2016-12-24 NP
Eur Wig/NL-Leidschendam/16015697-007/2016 2016-12-10 NP
Eur Wig/NL-Zoeterwoude/16015702-010/2016 2016-12-10 NP
Eur Wig/NL-West Graftdijk/16015746-003/2016 2016-12-12 NP
Eur Wig/NL-Vianen/16015917-006/2016 2016-12-13 NP
Eur Wig/NL-Gouda/16015824-001/2016 2016-12-13 NP
Eur Wig/NL-De Waal (Texel)/16014891-003/2016 2016-11-27 NP
Dk/NL-Stolwijk/16016291-016-020/2016 2016-12-21 NP
Dk/NL-Kamperveen/16016104-001-005/2016 2016-12-15 NP
A/ty/England/052131/2016) EPI ISL 239801 (H5N8) 2016-12-15 NP
Eur Wig/NL-Reeuwijk/16015903-003/2016 2016-12-13 NP
Ch/NL-Rhenen/16016141-006/2016 2016-12-17 NP
A/w dk/Tatarstan/3059/2016 EPI ISL 247724 (H5N8) 2016-10-02 NP
Eur Wig/NL-Greonterp/16015653-001/2016 2016-12-08 NP
A/dec dk/France/161104e/2016 EPI ISL 239997 (H5N8) 2016-11-26 NP
Eur Wig/NL-Drieborg (Dollard)/16015513-001/2016 2016-12-06 NP
Eur Wig/NL-Enumatil-Groningen/16015704-001/2016 2016-12-11 NP
Eur Wig/NL-Groningen/16015376-003/2016 2016-12-05 NP
Eur Wig/NL-Akkrum/16015817-003/2016 2016-12-13 NP
Mal/NL-Mastenbroek/16015378-002/2016 2016-12-05 NP
Eur Wig/NL-Walterswald/16015923-003/2016 2016-12-14 NP
Eur Wig/NL-Wormer/16016143-002/2016 2016-12-18 NP
Eur Wig/NL-Ferwert/16015273-002/2016 2016-12-01 NP
Teal/NL-Ferwert/16015273-013/2016 2016-12-01 NP
Ch/NL-Abbega/X16015736/2016 2016-12-12 NP
Ch/NL-Hiaure/16016112-001-005/2016 2016-12-17 NP
Eur Wig/NL-Leeuwarden/16015699-002/2016 2016-12-10 NP
Eur Wig/NL-Terschelling/16015692-010/2016 2016-12-09 NP
Crow/NL-Oosterwoud/16015372-004/2016 2016-12-05 NP
Magpie/NL-Volendam/16014331-002/2016 2016-11-16 NP
L-bl-ba-gull/NL-Sovon/16014324-014/2016 2016-11-16 NP
Buzard/NL-Durgerdam/16015100-004/2016 2016-11-30 NP
T Dk/NL-Werkendam/16014159-001/2016 2016-11-14 NP
Sea eagle/NL-Assen/16015398-002/2016 2016-12-05 NP
G c grebe/NL-Monnickendam/16013865-009-010/2016 2016-11-08 NP
A/w dk/Poland/82/2016 EPI ISL 237921 (H5N8) 2016-11-02 NP
Dk/NL-Biddinghuizen/16015083-016-020/2016 2016-11-30 NP
Dk/NL-Biddinghuizen/16015145-021-025/2016 2016-12-01 NP
Dk/NL-Biddinghuizen/16014829-011-015/2016 2016-11-25 NP
T Dk/NL-Roggebotsluis/16014462-015/2016 2016-11-17 NP
Gr bk bd gull/NL-Slootdorp/16014102-005/2016 2016-11-11 NP
C Gull/NL-Slootdorp/16014102-003/2016 2016-11-11 NP
Ch/NL-Den Dolder/17000410-011-015/2016 2017-01-10 NP
T Dk/NL-Monnickendam/16013865-006-008/2016 2016-11-08 NP
Go/NL-Roggebotsluis/16014462-010/2016 2016-11-17 NP
Bl H gull/NL-Slootdorp/16014102-002/2016 2016-11-11 NP
A/Ch/Sweden/SVA161122KU0453/SZ0209316/2016 EPI ISL 238893 (H5N8) 2016-11-21 NP
P falcon/NL-Vrouwenpolder (Zeeland)/16015510-001/2016 2016-12-07 NP
Dk /NL-Rotterdam/16014008-001-005/2016 2016-11-10 NP
Ch/NL-Den Oever/16014231-001/2016 2016-11-15 NP
Gull1/NL-Marker Wadden/16014466-011/2016 2016-11-17 NP
Bk swan/NL-Den Oever/16013973-002/2016 2016-11-10 NP
T Dk/NL-Rotterdam/16014155-001/2016 2016-11-14 NP
T Dk/NL-Almeerder Zand/16014341-003/2016 2016-11-16 NP
A/C Goldeneye/Sweden/SVA161117KU0322/SZ0002165/2016 EPI ISL 238897 (H5N8) 2016-11-14 NP
A/t dk/Germany/AR8444-L01986/2016 EPI ISL 237733-(H5N8) 2016-11-07 NP
T Dk/NL-Zeewolde/16013976-001/2016 2016-11-09 NP
T Dk/NL-Zuidoost Beemster/16014148-002/2016 2016-11-14 NP
M Swan/NL-Roggebotsluis/16014462-019/2016 2016-11-17 NP
A/dk/France/161108h/2016 EPI ISL 240012 (H5N8) 2016-11-28 NP
A/m swan/Croatia/70/2016 EPI ISL 238196 (H5N8) 2016-10-30 NP
Eurasia 2016
A/mal/Rep of Georgia/13/2011 EPI ISL 189700 (H6N2) 2011-11-26 NP
A/mal dk/Netherlands/12/2012 EPI ISL 243384 (H6N1) 2012-10-18 NP
A/ch/England/2830/2015 EPI ISL 171721 (H7N7) 2015-01-30 NP
A/mal dk/Netherlands/23/2012 EPI ISL 243461 (H11N9) 2012-09-27 NP
A/dk/Mongolia/173/2015 EPI ISL 209119 (H3N8) 2015-08-30 NP
A/dk/Guangxi/125D17/2012 EPI ISL 163619 (H4N2) 2012-09-07 NP
A/pa stork/India/10CA03/2016 EPI ISL 237554 (H5N8) | 2016-10-20 NP
A/pigeon/Egypt/S10409A/2014 EPI ISL 229599 (H9N2) 2014-12-08-09 NP
A/ch/Scotland/532/2016 EPI ISL 205833 (H5N1) 2016-01-09 NP
A/mal dk/Netherlands/7/2014 EPI ISL 243620 (H6N2) 2014-10-01 NP
A/ch/Astrakhan/3131/2016 EPI ISL 240110 (H5N8) 2016-12-13 NP
A/dom dk/Siberia/49 feather/2016 EPI ISL 250238 (H6N8) 2016-xx-xx NP
A/env/Kamchatka/18/2016 EPI ISL 256301 (H5N5) 2016-10-01 NP
M swan/NL-Groningen/16015826-001/2016 2016-12-13 NP
A/dk/Mongolia/200/2015 EPI ISL 209123 (H3N8) 2015-08-31 NP
A/wild waterfowl/Dongting/C2383/2012 EPI ISL 151927 (H1N2) 2012-01-10 NP
A/dk/Mongolia/211/2015 EPI ISL 209125 (H3N8) 2015-08-31 NP
A/dk/Mongolia/996/2015 EPI ISL 209135 (H3N8) 2015-08-31 NP
A/b-h Go/Qinghai/BTY2-LU/2016 EPI ISL 224708 (H5N8) 2016-05-12 NP
A/b-h Go/Qinghai/BTY3-B/2016 EPI ISL 224709 (H5N8) 2016-05-12 NP
A/b-h Go/Qinghai/BTY9-B/2016 EPI ISL 224721 (H5N8) 2016-05-09 NP
A/b-h Go/Qinghai/BTY1-B/2016 EPI ISL 224704 (H5N8) 2016-05-09 NP
A/b-h Go/Qinghai/BTY7-B/2016 EPI ISL 224716 (H5N8) 2016-05-11 NP
A/g c grebe/Tyvia/34/2016 EPI ISL 230820 (H5N8) 2016-05-25 NP
A/w dk/Tyvia/35/2016 EPI ISL 231684 (H5N8) 2016-05-25 NP
Russia/Mongolia 2016
A/dk/France/150236/2015 EPI ISL 234920 (H5N9) 2015-12-07 NP
A/go/Zhejiang/925037/2014 EPI ISL 203752 (H5N8) 2014-09-25 NP
A/b t/Korea/H52/2014 EPI ISL 171695 (H5N8) 2014-01-20 NP
A/dk/Eastern China/S1109/2014 EPI ISL 208841 (H5N8) 2014-11-09 NP
A/ch/California/15-004912/2015 EPI ISL 206415 (H5N8) 2015-02-06 NP
A/per fal/Washington/196426/2014 EPI ISL 206445 (H5N8) 2014-12-29 NP
A/mal/Oregon/195536/2014 EPI ISL 206441 (H5N8) 2014-12-24 NP
A/ty/California/K1500169-1.2/2015 EPI ISL 177647 (H5N8) 2015-01-21 NP
N. America 2014-15
A/b t/Korea/H96/2014 EPI ISL 171704 (H5N8) 2014-01-23 NP
A/ch/Miyazaki/2-4/2014 EPI ISL 199146 (H5N8)MP 2014-12-28 NP
A/b t/Korea/Donglim3/2014 EPI ISL 157611 (H5N8) 2014-01-17 NP
A/ch/Netherlands/14015531/2014 EPI ISL 168075 (H5N8) 2014-11-15 NP
A/dk/England/36254/14 EPI ISL 167904 (H5N8) 2014-11-14 NP
A/dk/Chiba/26-372-48/2014 EPI ISL 168024 (H5N8) 2014-11-18 NP
A/dk/Netherlands/14015898/2014 EPI ISL 174351 (H5N8) 2014-11-21 NP
Eurasia 2014
0.01

F

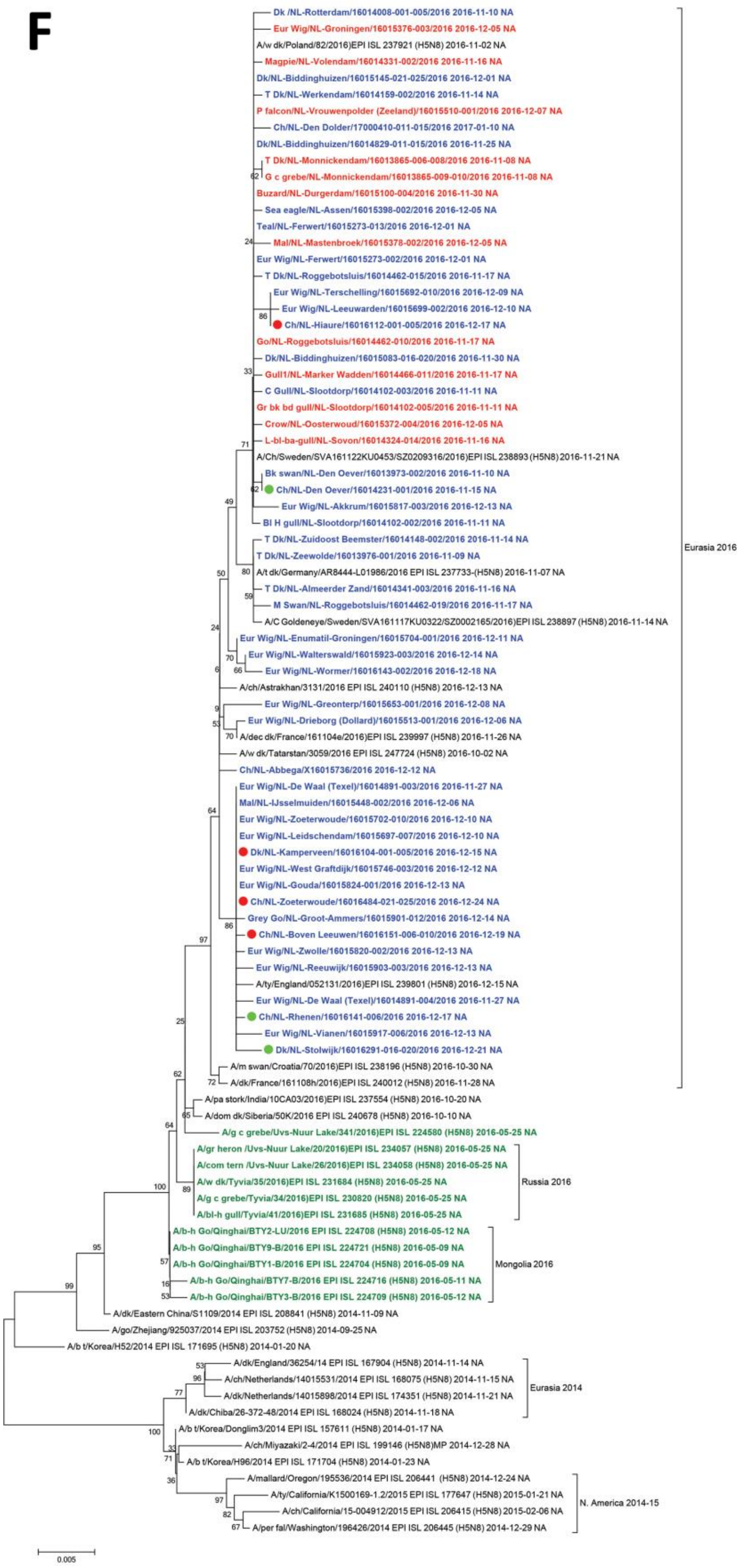
Dk /NL-Rotterdam/16014008-001-005/2016 2016-11-10 NA
Eur Wig/NL-Groningen/16015376-003/2016 2016-12-05 NA
A/w dk/Poland/82/2016)EPI ISL 237921 (H5N8) 2016-11-02 NA
Magpie/NL-Volendam/16014331-002/2016 2016-11-16 NA
Dk/NL-Biddinghuizen/16015145-021-025/2016 2016-12-01 NA
T Dk/NL-Werkendam/16014159-002/2016 2016-11-14 NA
P falcon/NL-Vrouwenpolder (Zeeland)/16015510-001/2016 2016-12-07 NA
Ch/NL-Den Dolder/17000410-011-015/2016 2017-01-10 NA
Dk/NL-Biddinghuizen/16014829-011-015/2016 2016-11-25 NA
T Dk/NL-Monnickendam/16013865-006-008/2016 2016-11-08 NA
G c grebe/NL-Monnickendam/16013865-009-010/2016 2016-11-08 NA
Buzard/NL-Durgerdam/16015100-004/2016 2016-11-30 NA
Sea eagle/NL-Assen/16015398-002/2016 2016-12-05 NA
Teal/NL-Ferwert/16015273-013/2016 2016-12-01 NA
Mal/NL-Mastenbroek/16015378-002/2016 2016-12-05 NA
Eur Wig/NL-Ferwert/16015273-002/2016 2016-12-01 NA
T Dk/NL-Roggebotsluis/16014462-015/2016 2016-11-17 NA
Eur Wig/NL-Terschelling/16015692-010/2016 2016-12-09 NA
Eur Wig/NL-Leeuwarden/16015699-002/2016 2016-12-10 NA
Ch/NL-Hiaure/16016112-001-005/2016 2016-12-17 NA
Go/NL-Roggebotsluis/16014462-010/2016 2016-11-17 NA
Dk/NL-Biddinghuizen/16015083-016-020/2016 2016-11-30 NA
Gull1/NL-Marker Wadden/16014466-011/2016 2016-11-17 NA
C Gull/NL-Slootdorp/16014102-003/2016 2016-11-11 NA
Gr bk bd gull/NL-Slootdorp/16014102-005/2016 2016-11-11 NA
Crow/NL-Oosterwoud/16015372-004/2016 2016-12-05 NA
L-bl-ba-gull/NL-Sovon/16014324-014/2016 2016-11-16 NA
A/Ch/Sweden/SVA161122KU0453/SZ0209316/2016)EPI ISL 238893 (H5N8) 2016-11-21 NA
Bk swan/NL-Den Oever/16013973-002/2016 2016-11-10 NA
Ch/NL-Den Oever/16014231-001/2016 2016-11-15 NA
Eur Wig/NL-Akkrum/16015817-003/2016 2016-12-13 NA
Bl H gull/NL-Slootdorp/16014102-002/2016 2016-11-11 NA
T Dk/NL-Zuidoost Beemster/16014148-002/2016 2016-11-14 NA
T Dk/NL-Zeewolde/16013976-001/2016 2016-11-09 NA
A/t dk/Germany/AR8444-L01986/2016 EPI ISL 237733-(H5N8) 2016-11-07 NA
T Dk/NL-Almeerder Zand/16014341-003/2016 2016-11-16 NA
M Swan/NL-Roggebotsluis/16014462-019/2016 2016-11-17 NA
A/C Goldeneye/Sweden/SVA161117KU0322/SZ0002165/2016)EPI ISL 238897 (H5N8) 2016-11-14 NA
Eur Wig/NL-Enumatil-Groningen/16015704-001/2016 2016-12-11 NA
Eur Wig/NL-Walterswald/16015923-003/2016 2016-12-14 NA
Eur Wig/NL-Wormer/16016143-002/2016 2016-12-18 NA
A/ch/Astrakhan/3131/2016 EPI ISL 240110 (H5N8) 2016-12-13 NA
Eur Wig/NL-Greonterp/16015653-001/2016 2016-12-08 NA
Eur Wig/NL-Drieborg (Dollard)/16015513-001/2016 2016-12-06 NA
A/dec dk/France/161104e/2016)EPI ISL 239997 (H5N8) 2016-11-26 NA
A/w dk/Tatarstan/3059/2016 EPI ISL 247724 (H5N8) 2016-10-02 NA
Ch/NL-Abbega/X16015736/2016 2016-12-12 NA
Eur Wig/NL-De Waal (Texel)/16014891-003/2016 2016-11-27 NA
Mal/NL-IJsselmuiden/16015448-002/2016 2016-12-06 NA
Eur Wig/NL-Zoeterwoude/16015702-010/2016 2016-12-10 NA
Eur Wig/NL-Leidschendam/16015697-007/2016 2016-12-10 NA
Dk/NL-Kamperveen/16016104-001-005/2016 2016-12-15 NA
Eur Wig/NL-West Graftdijk/16015746-003/2016 2016-12-12 NA
Eur Wig/NL-Gouda/16015824-001/2016 2016-12-13 NA
Ch/NL-Zoeterwoude/16016484-021-025/2016 2016-12-24 NA
Grey Go/NL-Groot-Ammers/16015901-012/2016 2016-12-14 NA
Ch/NL-Boven Leeuwen/16016151-006-010/2016 2016-12-19 NA
Eur Wig/NL-Zwolle/16015820-002/2016 2016-12-13 NA
Eur Wig/NL-Reeuwijk/16015903-003/2016 2016-12-13 NA
A/ty/England/052131/2016)EPI ISL 239801 (H5N8) 2016-12-15 NA
Eur Wig/NL-De Waal (Texel)/16014891-004/2016 2016-11-27 NA
Ch/NL-Rhenen/16016141-006/2016 2016-12-17 NA
Eur Wig/NL-Vianen/16015917-006/2016 2016-12-13 NA
Dk/NL-Stolwijk/16016291-016-020/2016 2016-12-21 NA
A/m swan/Croatia/70/2016)EPI ISL 238196 (H5N8) 2016-10-30 NA
A/dk/France/161108h/2016)EPI ISL 240012 (H5N8) 2016-11-28 NA
Eurasia 2016
A/pa stork/India/10CA03/2016)EPI ISL 237554 (H5N8) 2016-10-20 NA
A/dom dk/Siberia/50K/2016 EPI ISL 240678 (H5N8) 2016-10-10 NA
A/g c grebe/Uvs-Nuur Lake/341/2016)EPI ISL 224580 (H5N8) 2016-05-25 NA
A/gr heron /Uvs-Nuur Lake/20/2016)EPI ISL 234057 (H5N8) 2016-05-25 NA
A/com tern /Uvs-Nuur Lake/26/2016)EPI ISL 234058 (H5N8) 2016-05-25 NA
A/w dk/Tyvia/35/2016)EPI ISL 231684 (H5N8) 2016-05-25 NA
A/g c grebe/Tyvia/34/2016)EPI ISL 230820 (H5N8) 2016-05-25 NA
A/bl-h gull/Tyvia/41/2016)EPI ISL 231685 (H5N8) 2016-05-25 NA
Russia 2016
A/b-h Go/Qinghai/BTY2-LU/2016 EPI ISL 224708 (H5N8) 2016-05-12 NA
A/b-h Go/Qinghai/BTY9-B/2016 EPI ISL 224721 (H5N8) 2016-05-09 NA
A/b-h Go/Qinghai/BTY1-B/2016 EPI ISL 224704 (H5N8) 2016-05-09 NA
A/b-h Go/Qinghai/BTY7-B/2016 EPI ISL 224716 (H5N8) 2016-05-11 NA
A/b-h Go/Qinghai/BTY3-B/2016 EPI ISL 224709 (H5N8) 2016-05-12 NA
Mongolia 2016
A/dk/Eastern China/S1109/2014 EPI ISL 208841 (H5N8) 2014-11-09 NA
A/go/Zhejiang/925037/2014 EPI ISL 203752 (H5N8) 2014-09-25 NA
A/b t/Korea/H52/2014 EPI ISL 171695 (H5N8) 2014-01-20 NA
A/dk/England/36254/14 EPI ISL 167904 (H5N8) 2014-11-14 NA
A/ch/Netherlands/14015531/2014 EPI ISL 168075 (H5N8) 2014-11-15 NA
A/dk/Netherlands/14015898/2014 EPI ISL 174351 (H5N8) 2014-11-21 NA
A/dk/Chiba/26-372-48/2014 EPI ISL 168024 (H5N8) 2014-11-18 NA
Eurasia 2014
A/b t/Korea/Donglim3/2014 EPI ISL 157611 (H5N8) 2014-01-17 NA
A/ch/Miyazaki/2-4/2014 EPI ISL 199146 (H5N8)MP 2014-12-28 NA
A/b t/Korea/H96/2014 EPI ISL 171704 (H5N8) 2014-01-23 NA
A/mallard/Oregon/195536/2014 EPI ISL 206441 (H5N8) 2014-12-24 NA
A/ty/California/K1500169-1.2/2015 EPI ISL 177647 (H5N8) 2015-01-21 NA
A/ch/California/15-004912/2015 EPI ISL 206415 (H5N8) 2015-02-06 NA
A/per fal/Washington/196426/2014 EPI ISL 206445 (H5N8) 2014-12-29 NA
N. America 2014-15
0.005

G

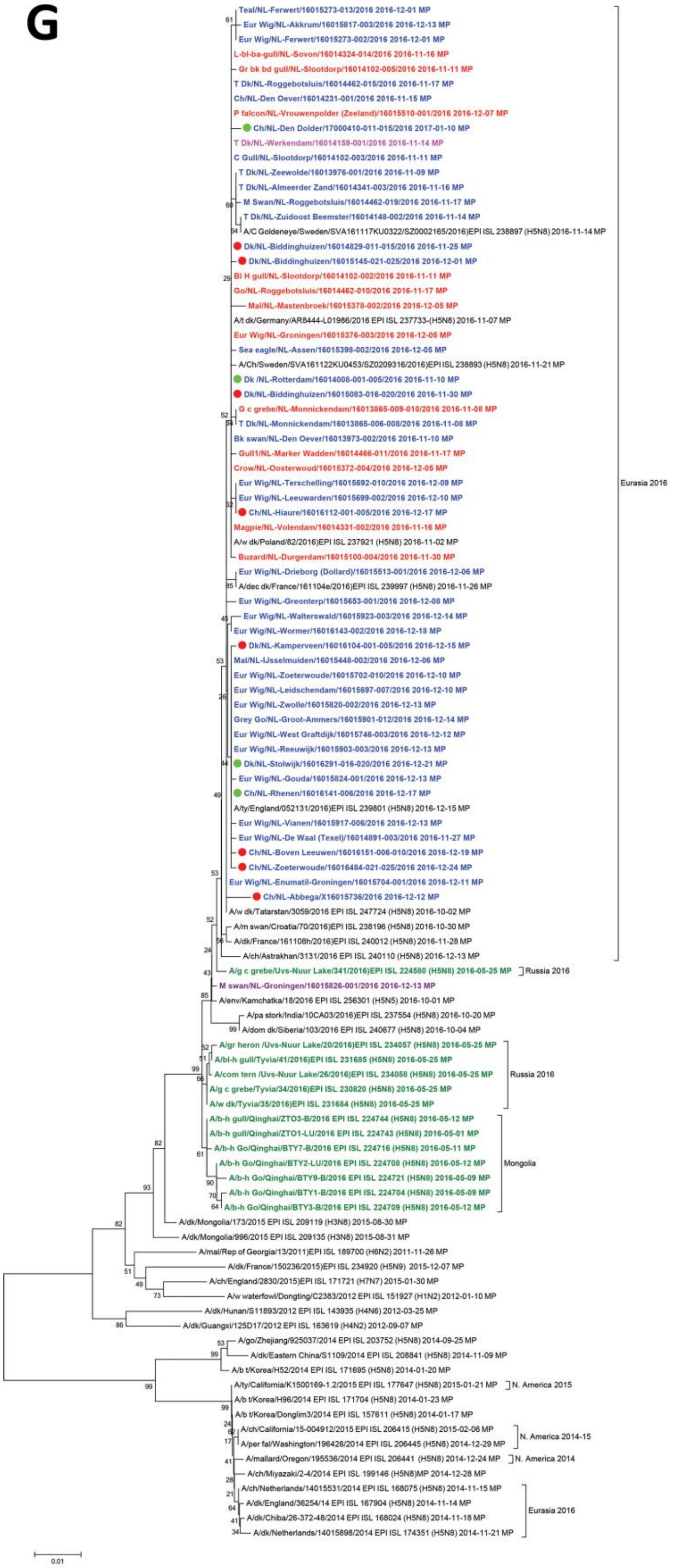

Teal/NL-Ferwert/16015273-013/2016 2016-12-01 MP
Eur Wig/NL-Akkrum/16015817-003/2016 2016-12-13 MP
Eur Wig/NL-Ferwert/16015273-002/2016 2016-12-01 MP
L-bl-ba-gull/NL-Sovon/16014324-014/2016 2016-11-16 MP
Gr bk bd gull/NL-Slootdorp/16014102-005/2016 2016-11-11 MP
T Dk/NL-Roggebotsluis/16014462-015/2016 2016-11-17 MP
Ch/NL-Den Oever/16014231-001/2016 2016-11-15 MP
P falcon/NL-Vrouwenpolder (Zeeland)/16015510-001/2016 2016-12-07 MP
Ch/NL-Den Dolder/17000410-011-015/2016 2017-01-10 MP
T Dk/NL-Werkendam/16014159-001/2016 2016-11-14 MP
C Gull/NL-Slootdorp/16014102-003/2016 2016-11-11 MP
T Dk/NL-Zeewolde/16013976-001/2016 2016-11-09 MP
T Dk/NL-Almeerder Zand/16014341-003/2016 2016-11-16 MP
M Swan/NL-Roggebotsluis/16014462-019/2016 2016-11-17 MP
T Dk/NL-Zuidoost Beemster/16014148-002/2016 2016-11-14 MP
A/C Goldeneye/Sweden/SVA161117KU0322/SZ0002165/2016)EPI ISL 238897 (H5N8) 2016-11-14 MP
Dk/NL-Biddinghuizen/16014829-011-015/2016 2016-11-25 MP
Dk/NL-Biddinghuizen/16015145-021-025/2016 2016-12-01 MP
Bl H gull/NL-Slootdorp/16014102-002/2016 2016-11-11 MP
Go/NL-Roggebotsluis/16014462-010/2016 2016-11-17 MP
Mal/NL-Mastenbroek/16015378-002/2016 2016-12-05 MP
A/t dk/Germany/AR8444-L01986/2016 EPI ISL 237733-(H5N8) 2016-11-07 MP
Eur Wig/NL-Groningen/16015376-003/2016 2016-12-05 MP
Sea eagle/NL-Assen/16015398-002/2016 2016-12-05 MP
A/Ch/Sweden/SVA161122KU0453/SZ0209316/2016)EPI ISL 238893 (H5N8) 2016-11-21 MP
Dk /NL-Rotterdam/16014008-001-005/2016 2016-11-10 MP
Dk/NL-Biddinghuizen/16015083-016-020/2016 2016-11-30 MP
G c grebe/NL-Monnickendam/16013865-009-010/2016 2016-11-08 MP
T Dk/NL-Monnickendam/16013865-006-008/2016 2016-11-08 MP
Bk swan/NL-Den Oever/16013973-002/2016 2016-11-10 MP
Gull1/NL-Marker Wadden/16014466-011/2016 2016-11-17 MP
Crow/NL-Oosterwoud/16015372-004/2016 2016-12-05 MP
Eur Wig/NL-Terschelling/16015692-010/2016 2016-12-09 MP
Eur Wig/NL-Leeuwarden/16015699-002/2016 2016-12-10 MP
Ch/NL-Hiaure/16016112-001-005/2016 2016-12-17 MP
Magpie/NL-Volendam/16014331-002/2016 2016-11-16 MP
A/w dk/Poland/82/2016)EPI ISL 237921 (H5N8) 2016-11-02 MP
Buzard/NL-Durgerdam/16015100-004/2016 2016-11-30 MP
Eur Wig/NL-Drieborg (Dollard)/16015513-001/2016 2016-12-06 MP
A/dec dk/France/161104e/2016)EPI ISL 239997 (H5N8) 2016-11-26 MP
Eur Wig/NL-Greonterp/16015653-001/2016 2016-12-08 MP
Eur Wig/NL-Walterswald/16015923-003/2016 2016-12-14 MP
Eur Wig/NL-Wormer/16016143-002/2016 2016-12-18 MP
Dk/NL-Kamperveen/16016104-001-005/2016 2016-12-15 MP
Mal/NL-IJsselmuiden/16015448-002/2016 2016-12-06 MP
Eur Wig/NL-Zoeterwoude/16015702-010/2016 2016-12-10 MP
Eur Wig/NL-Leidschendam/16015697-007/2016 2016-12-10 MP
Eur Wig/NL-Zwolle/16015820-002/2016 2016-12-13 MP
Grey Go/NL-Groot-Ammers/16015901-012/2016 2016-12-14 MP
Eur Wig/NL-West Graftdijk/16015746-003/2016 2016-12-12 MP
Eur Wig/NL-Reeuwijk/16015903-003/2016 2016-12-13 MP
Dk/NL-Stolwijk/16016291-016-020/2016 2016-12-21 MP
Eur Wig/NL-Gouda/16015824-001/2016 2016-12-13 MP
Ch/NL-Rhenen/16016141-006/2016 2016-12-17 MP
A/ty/England/052131/2016)EPI ISL 239801 (H5N8) 2016-12-15 MP
Eur Wig/NL-Vianen/16015917-006/2016 2016-12-13 MP
Eur Wig/NL-De Waal (Texel)/16014891-003/2016 2016-11-27 MP
Ch/NL-Boven Leeuwen/16016151-006-010/2016 2016-12-19 MP
Ch/NL-Zoeterwoude/16016484-021-025/2016 2016-12-24 MP
Eur Wig/NL-Enumatil-Groningen/16015704-001/2016 2016-12-11 MP
Ch/NL-Abbega/X16015736/2016 2016-12-12 MP
A/w dk/Tatarstan/3059/2016 EPI ISL 247724 (H5N8) 2016-10-02 MP
A/m swan/Croatia/70/2016)EPI ISL 238196 (H5N8) 2016-10-30 MP
A/dk/France/161108h/2016)EPI ISL 240012 (H5N8) 2016-11-28 MP
A/ch/Astrakhan/3131/2016 EPI ISL 240110 (H5N8) 2016-12-13 MP
Eurasia 2016
A/g c grebe/Uvs-Nuur Lake/341/2016)EPI ISL 224580 (H5N8) 2016-05-25 MP
Russia 2016
M swan/NL-Groningen/16015826-001/2016 2016-12-13 MP
A/env/Kamchatka/18/2016 EPI ISL 256301 (H5N5) 2016-10-01 MP
A/pa stork/India/10CA03/2016)EPI ISL 237554 (H5N8) 2016-10-20 MP
A/dom dk/Siberia/103/2016 EPI ISL 240677 (H5N8) 2016-10-04 MP
A/gr heron /Uvs-Nuur Lake/20/2016)EPI ISL 234057 (H5N8) 2016-05-25 MP
A/bl-h gull/Tyvia/41/2016)EPI ISL 231685 (H5N8) 2016-05-25 MP
A/com tern /Uvs-Nuur Lake/26/2016)EPI ISL 234058 (H5N8) 2016-05-25 MP
A/g c grebe/Tyvia/34/2016)EPI ISL 230820 (H5N8) 2016-05-25 MP
A/w dk/Tyvia/35/2016)EPI ISL 231684 (H5N8) 2016-05-25 MP
Russia 2016
A/b-h gull/Qinghai/ZTO3-B/2016 EPI ISL 224744 (H5N8) 2016-05-12 MP
A/b-h gull/Qinghai/ZTO1-LU/2016 EPI ISL 224743 (H5N8) 2016-05-01 MP
A/b-h Go/Qinghai/BTY7-B/2016 EPI ISL 224716 (H5N8) 2016-05-11 MP
A/b-h Go/Qinghai/BTY2-LU/2016 EPI ISL 224708 (H5N8) 2016-05-12 MP
A/b-h Go/Qinghai/BTY9-B/2016 EPI ISL 224721 (H5N8) 2016-05-09 MP
A/b-h Go/Qinghai/BTY1-B/2016 EPI ISL 224704 (H5N8) 2016-05-09 MP
A/b-h Go/Qinghai/BTY3-B/2016 EPI ISL 224709 (H5N8) 2016-05-12 MP
Mongolia
A/dk/Mongolia/173/2015 EPI ISL 209119 (H3N8) 2015-08-30 MP
A/dk/Mongolia/996/2015 EPI ISL 209135 (H3N8) 2015-08-31 MP
A/mal/Rep of Georgia/13/2011)EPI ISL 189700 (H6N2) 2011-11-26 MP
A/dk/France/150236/2015)EPI ISL 234920 (H5N9) 2015-12-07 MP
A/ch/England/2830/2015)EPI ISL 171721 (H7N7) 2015-01-30 MP
A/w waterfowl/Dongting/C2383/2012 EPI ISL 151927 (H1N2) 2012-01-10 MP
A/dk/Hunan/S11893/2012 EPI ISL 143935 (H4N6) 2012-03-25 MP
A/dk/Guangxi/125D17/2012 EPI ISL 163619 (H4N2) 2012-09-07 MP
A/go/Zhejiang/925037/2014 EPI ISL 203752 (H5N8) 2014-09-25 MP
A/dk/Eastern China/S1109/2014 EPI ISL 208841 (H5N8) 2014-11-09 MP
A/b t/Korea/H52/2014 EPI ISL 171695 (H5N8) 2014-01-20 MP
A/ty/California/K1500169-1.2/2015 EPI ISL 177647 (H5N8) 2015-01-21 MP
N. America 2015
A/b t/Korea/H96/2014 EPI ISL 171704 (H5N8) 2014-01-23 MP
A/b t/Korea/Donglim3/2014 EPI ISL 157611 (H5N8) 2014-01-17 MP
A/ch/California/15-004912/2015 EPI ISL 206415 (H5N8) 2015-02-06 MP
A/per fal/Washington/196426/2014 EPI ISL 206445 (H5N8) 2014-12-29 MP
N. America 2014-15
A/mallard/Oregon/195536/2014 EPI ISL 206441 (H5N8) 2014-12-24 MP
N. America 2014
A/ch/Miyazaki/2-4/2014 EPI ISL 199146 (H5N8)MP 2014-12-28 MP
A/ch/Netherlands/14015531/2014 EPI ISL 168075 (H5N8) 2014-11-15 MP
A/dk/England/36254/14 EPI ISL 167904 (H5N8) 2014-11-14 MP
A/dk/Chiba/26-372-48/2014 EPI ISL 168024 (H5N8) 2014-11-18 MP
A/dk/Netherlands/14015898/2014 EPI ISL 174351 (H5N8) 2014-11-21 MP
Eurasia 2016
0.01

H

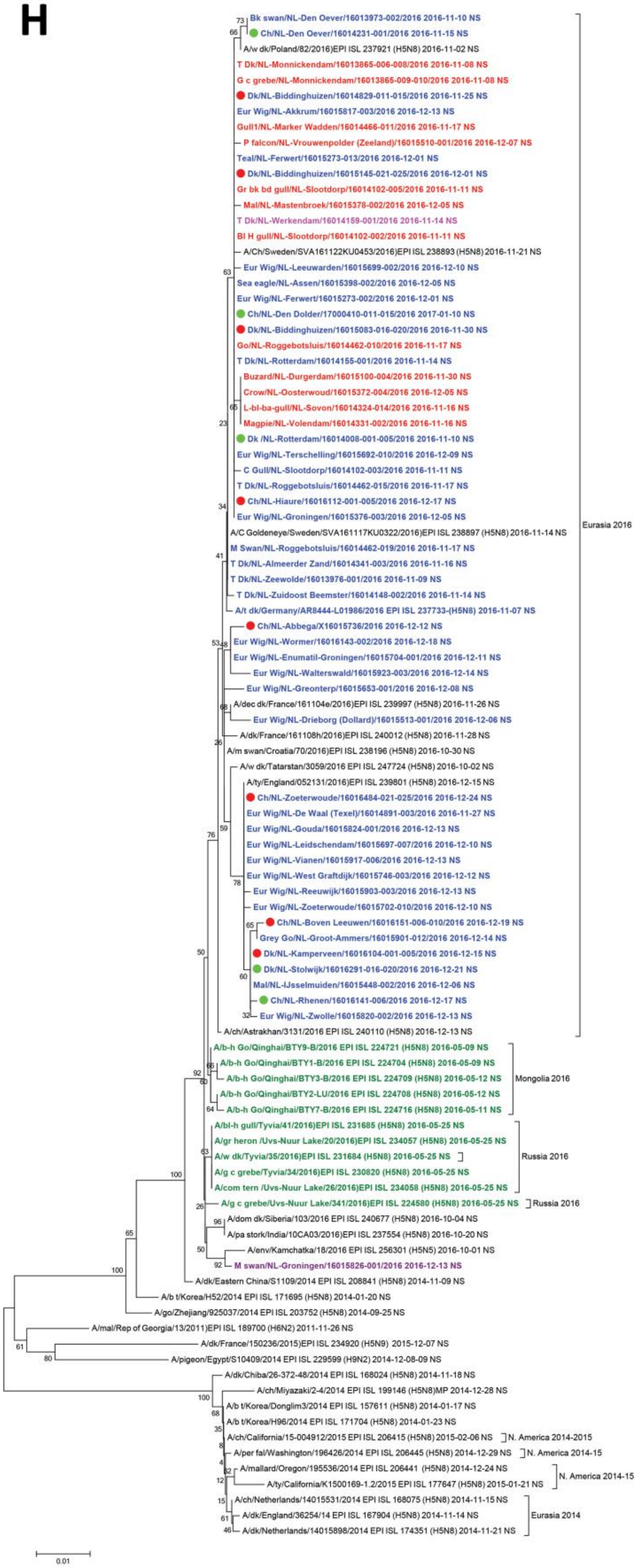
Eurasia 2016
Mongolia 2016
Russia 2016
N. America 2014-2015
N. America 2014-15
Eurasia 2014
0.01

I

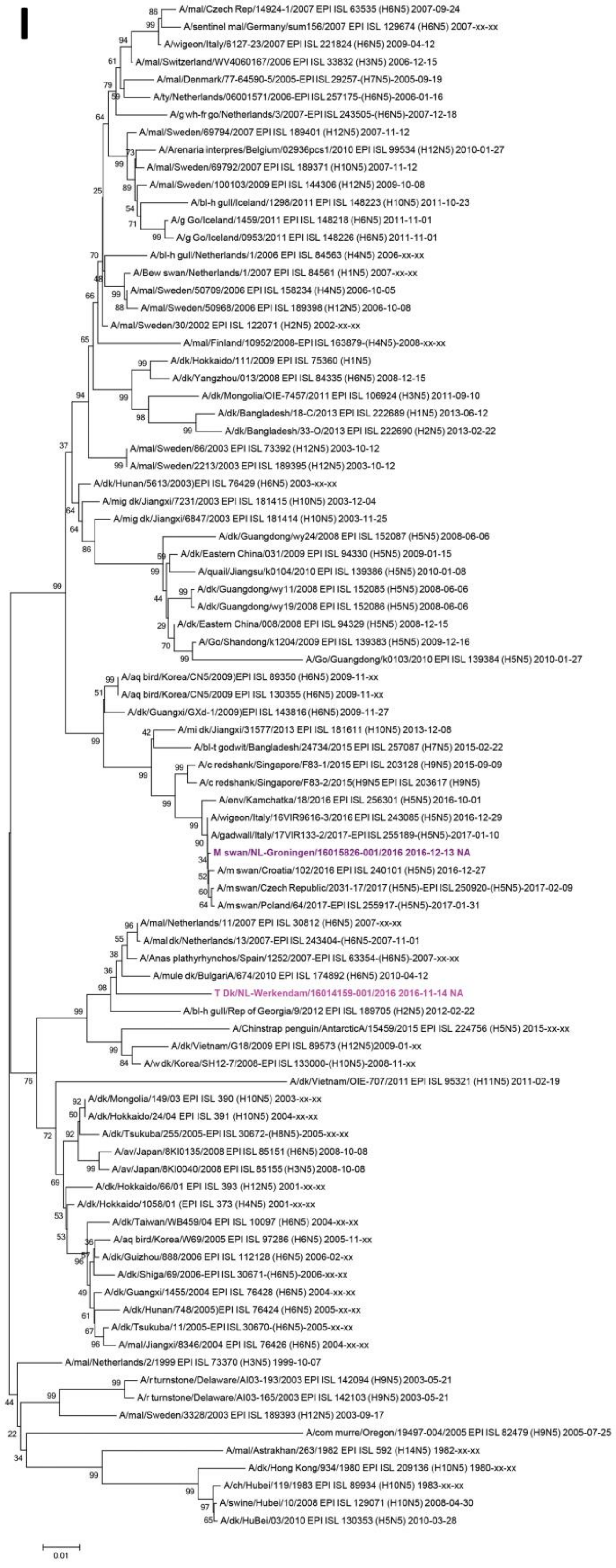

A/mal/Czech Rep/14924-1/2007 EPI ISL 63535 (H6N5) 2007-09-24
A/sentinel mal/Germany/sum156/2007 EPI ISL 129674 (H6N5) 2007-xx-xx
A/wigeon/Italy/6127-23/2007 EPI ISL 221824 (H6N5) 2009-04-12
A/mal/Switzerland/WV4060167/2006 EPI ISL 33832 (H3N5) 2006-12-15
A/mal/Denmark/77-64590-5/2005-EPI ISL 29257-(H7N5)-2005-09-19
A/ty/Netherlands/06001571/2006-EPI ISL 257175-(H6N5)-2006-01-16
A/g wh-fr go/Netherlands/3/2007-EPI ISL 243505-(H6N5)-2007-12-18
A/mal/Sweden/69794/2007 EPI ISL 189401 (H12N5) 2007-11-12
A/Arenaria interpres/Belgium/02936pcs1/2010 EPI ISL 99534 (H12N5) 2010-01-27
A/mal/Sweden/69792/2007 EPI ISL 189371 (H10N5) 2007-11-12
A/mal/Sweden/100103/2009 EPI ISL 144306 (H12N5) 2009-10-08
A/bl-h gull/Iceland/1298/2011 EPI ISL 148223 (H10N5) 2011-10-23
A/g Go/Iceland/1459/2011 EPI ISL 148218 (H6N5) 2011-11-01
A/g Go/Iceland/0953/2011 EPI ISL 148226 (H6N5) 2011-11-01
A/bl-h gull/Netherlands/1/2006 EPI ISL 84563 (H4N5) 2006-xx-xx
A/Bew swan/Netherlands/1/2007 EPI ISL 84561 (H1N5) 2007-xx-xx
A/mal/Sweden/50709/2006 EPI ISL 158234 (H4N5) 2006-10-05
A/mal/Sweden/50968/2006 EPI ISL 189398 (H12N5) 2006-10-08
A/mal/Sweden/30/2002 EPI ISL 122071 (H2N5) 2002-xx-xx
A/mal/Finland/10952/2008-EPI ISL 163879-(H4N5)-2008-xx-xx
A/dk/Hokkaido/111/2009 EPI ISL 75360 (H1N5)
A/dk/Yangzhou/013/2008 EPI ISL 84335 (H6N5) 2008-12-15
A/dk/Mongolia/OIE-7457/2011 EPI ISL 106924 (H3N5) 2011-09-10
A/dk/Bangladesh/18-C/2013 EPI ISL 222689 (H1N5) 2013-06-12
A/dk/Bangladesh/33-O/2013 EPI ISL 222690 (H2N5) 2013-02-22
A/mal/Sweden/86/2003 EPI ISL 73392 (H12N5) 2003-10-12
A/mal/Sweden/2213/2003 EPI ISL 189395 (H12N5) 2003-10-12
A/dk/Hunan/5613/2003)EPI ISL 76429 (H6N5) 2003-xx-xx
A/mig dk/Jiangxi/7231/2003 EPI ISL 181415 (H10N5) 2003-12-04
A/mig dk/Jiangxi/6847/2003 EPI ISL 181414 (H10N5) 2003-11-25
A/dk/Guangdong/wy24/2008 EPI ISL 152087 (H5N5) 2008-06-06
A/dk/Eastern China/031/2009 EPI ISL 94330 (H5N5) 2009-01-15
A/quail/Jiangsu/k0104/2010 EPI ISL 139386 (H5N5) 2010-01-08
A/dk/Guangdong/wy11/2008 EPI ISL 152085 (H5N5) 2008-06-06
A/dk/Guangdong/wy19/2008 EPI ISL 152086 (H5N5) 2008-06-06
A/dk/Eastern China/008/2008 EPI ISL 94329 (H5N5) 2008-12-15
A/Go/Shandong/k1204/2009 EPI ISL 139383 (H5N5) 2009-12-16
A/Go/Guangdong/k0103/2010 EPI ISL 139384 (H5N5) 2010-01-27
A/aq bird/Korea/CN5/2009)EPI ISL 89350 (H6N5) 2009-11-xx
A/aq bird/Korea/CN5/2009 EPI ISL 130355 (H6N5) 2009-11-xx
A/dk/Guangxi/GXd-1/2009)EPI ISL 143816 (H6N5) 2009-11-27
A/mi dk/Jiangxi/31577/2013 EPI ISL 181611 (H10N5) 2013-12-08
A/bl-t godwit/Bangladesh/24734/2015 EPI ISL 257087 (H7N5) 2015-02-22
A/c redshank/Singapore/F83-1/2015 EPI ISL 203128 (H9N5) 2015-09-09
A/c redshank/Singapore/F83-2/2015(H9N5 EPI ISL 203617 (H9N5)
A/env/Kamchatka/18/2016 EPI ISL 256301 (H5N5) 2016-10-01
A/wigeon/Italy/16VIR9616-3/2016 EPI ISL 243085 (H5N5) 2016-12-29
A/gadwall/Italy/17VIR133-2/2017-EPI ISL 255189-(H5N5)-2017-01-10
M swan/NL-Groningen/16015826-001/2016 2016-12-13 NA
A/m swan/Croatia/102/2016 EPI ISL 240101 (H5N5) 2016-12-27
A/m swan/Czech Republic/2031-17/2017 (H5N5)-EPI ISL 250920-(H5N5)-2017-02-09
A/m swan/Poland/64/2017-EPI ISL 255917-(H5N5)-2017-01-31
A/mal/Netherlands/11/2007 EPI ISL 30812 (H6N5) 2007-xx-xx
A/mal dk/Netherlands/13/2007-EPI ISL 243404-(H6N5-2007-11-01
A/Anas plathyrhynchos/Spain/1252/2007-EPI ISL 63354-(H6N5)-2007-xx-xx
A/mule dk/BulgariA/674/2010 EPI ISL 174892 (H6N5) 2010-04-12
T Dk/NL-Werkendam/16014159-001/2016 2016-11-14 NA
A/bl-h gull/Rep of Georgia/9/2012 EPI ISL 189705 (H2N5) 2012-02-22
A/Chinstrap penguin/AntarcticA/15459/2015 EPI ISL 224756 (H5N5) 2015-xx-xx
A/dk/Vietnam/G18/2009 EPI ISL 89573 (H12N5)2009-01-xx
A/w dk/Korea/SH12-7/2008-EPI ISL 133000-(H10N5)-2008-11-xx
A/dk/Vietnam/OIE-707/2011 EPI ISL 95321 (H11N5) 2011-02-19
A/dk/Mongolia/149/03 EPI ISL 390 (H10N5) 2003-xx-xx
A/dk/Hokkaido/24/04 EPI ISL 391 (H10N5) 2004-xx-xx
A/dk/Tsukuba/255/2005-EPI ISL 30672-(H8N5)-2005-xx-xx
A/av/Japan/8KI0135/2008 EPI ISL 85151 (H6N5) 2008-10-08
A/av/Japan/8KI0040/2008 EPI ISL 85155 (H3N5) 2008-10-08
A/dk/Hokkaido/66/01 EPI ISL 393 (H12N5) 2001-xx-xx
A/dk/Hokkaido/1058/01 (EPI ISL 373 (H4N5) 2001-xx-xx
A/dk/Taiwan/WB459/04 EPI ISL 10097 (H6N5) 2004-xx-xx
A/aq bird/Korea/W69/2005 EPI ISL 97286 (H6N5) 2005-11-xx
A/dk/Guizhou/888/2006 EPI ISL 112128 (H6N5) 2006-02-xx
A/dk/Shiga/69/2006-EPI ISL 30671-(H6N5)-2006-xx-xx
A/dk/Guangxi/1455/2004 EPI ISL 76428 (H6N5) 2004-xx-xx
A/dk/Hunan/748/2005)EPI ISL 76424 (H6N5) 2005-xx-xx
A/dk/Tsukuba/11/2005-EPI ISL 30670-(H6N5)-2005-xx-xx
A/mal/Jiangxi/8346/2004 EPI ISL 76426 (H6N5) 2004-xx-xx
A/mal/Netherlands/2/1999 EPI ISL 73370 (H3N5) 1999-10-07
A/r turnstone/Delaware/AI03-193/2003 EPI ISL 142094 (H9N5) 2003-05-21
A/r turnstone/Delaware/AI03-165/2003 EPI ISL 142103 (H9N5) 2003-05-21
A/mal/Sweden/3328/2003 EPI ISL 189393 (H12N5) 2003-09-17
A/com murre/Oregon/19497-004/2005 EPI ISL 82479 (H9N5) 2005-07-25
A/mal/Astrakhan/263/1982 EPI ISL 592 (H14N5) 1982-xx-xx
A/dk/Hong Kong/934/1980 EPI ISL 209136 (H10N5) 1980-xx-xx
A/ch/Hubei/119/1983 EPI ISL 89934 (H10N5) 1983-xx-xx
A/swine/Hubei/10/2008 EPI ISL 129071 (H10N5) 2008-04-30
A/dk/HuBei/03/2010 EPI ISL 130353 (H5N5) 2010-03-28
0.01

Technical Appendix 2 Figure 3. Molecular clock: Time-scaled phylogenies (dates shown on the horizontal axis) were inferred using relaxed-clock Bayesian Markov Chain Monte Carlo analysis for each gene segment separately (PB2, PB1, PA, HA, NP, N8, MP, NS) and also for N5. In this analysis, all sequences (mostly two, originating from the tracheal and cloacal swabs) obtained for the viruses isolated from wild birds, poultry or captive birds were included. The calculated time of most recent common ancestor (tMRCA) for the numbered nodes is listed in Table 1 in the main text), as are the highest posterior density interval and posterior value. The Russian-Mongolian H5N8 viruses are marked in green, the 2016 H5N8 viruses in the Netherlands are marked in blue (PA cluster I) and red (PA cluster II), and the H5N5 sequences isolated from a tufted duck near Werkendam (pink) and a mute swan near Groningen are also shown. The GISAID accession numbers of the viruses used in this study are listed in online Technical Appendix 1 Tables 1 and 2.

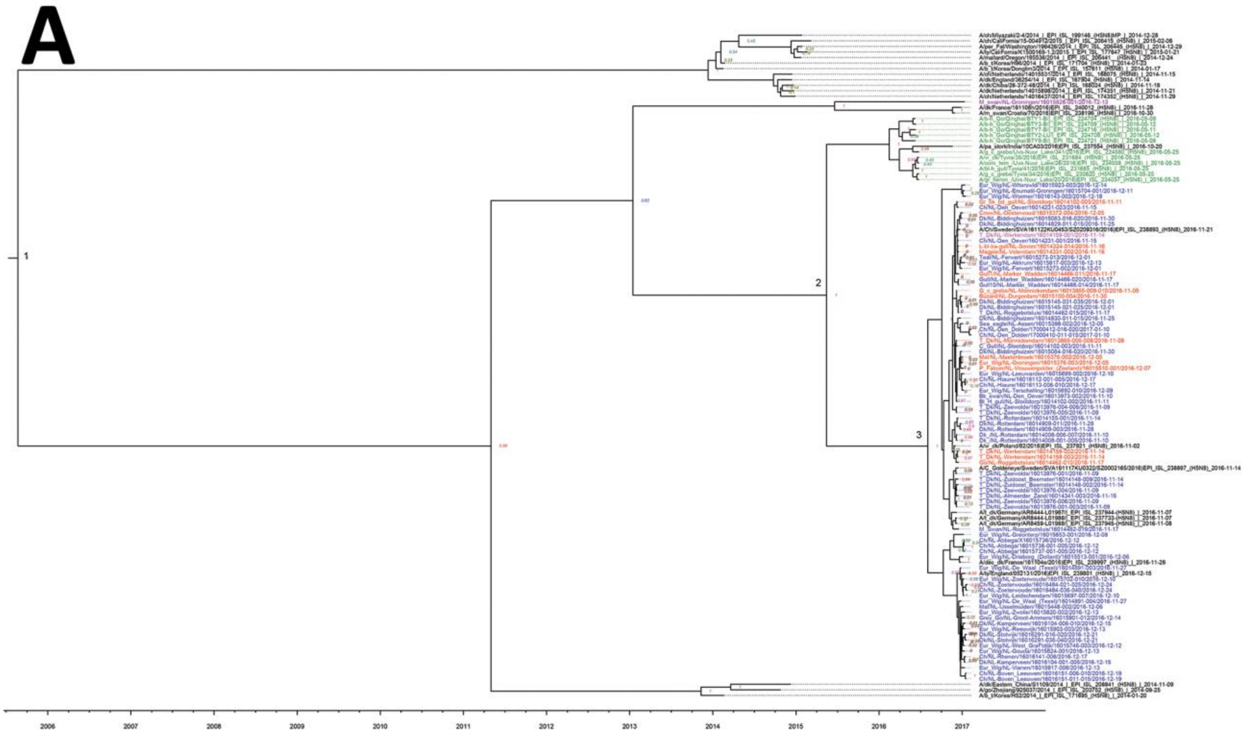

B

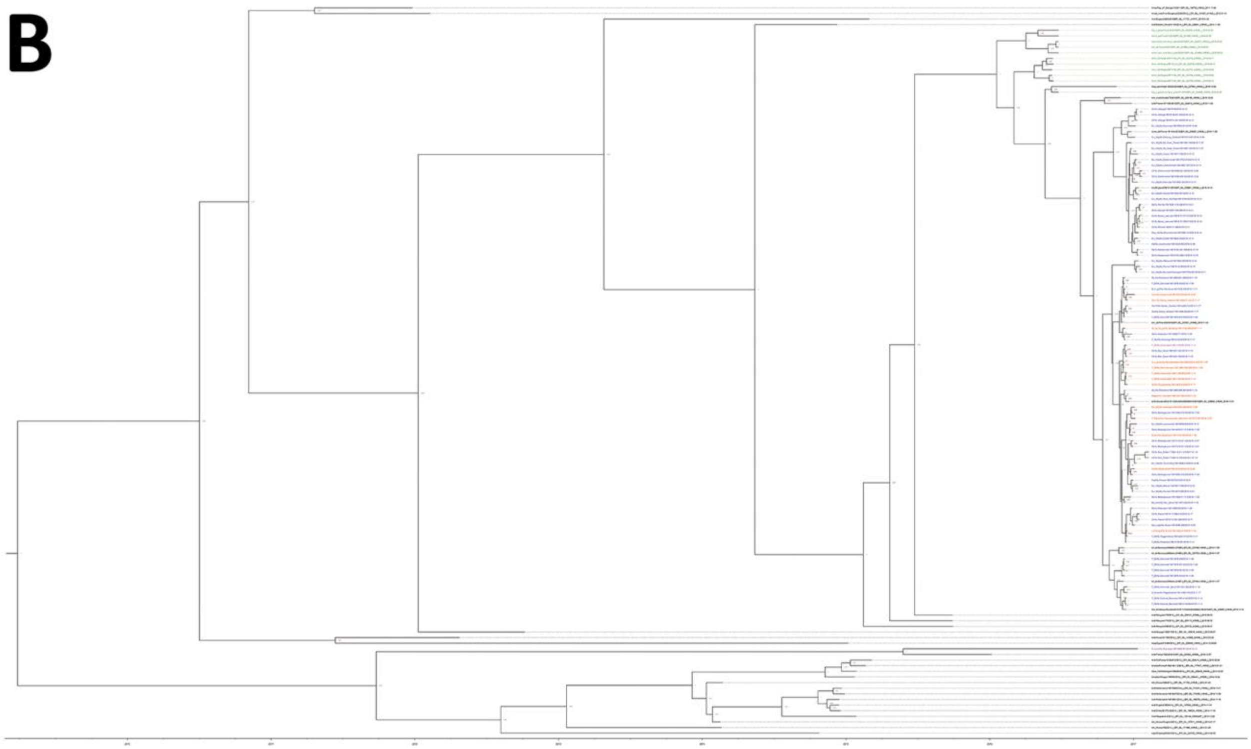

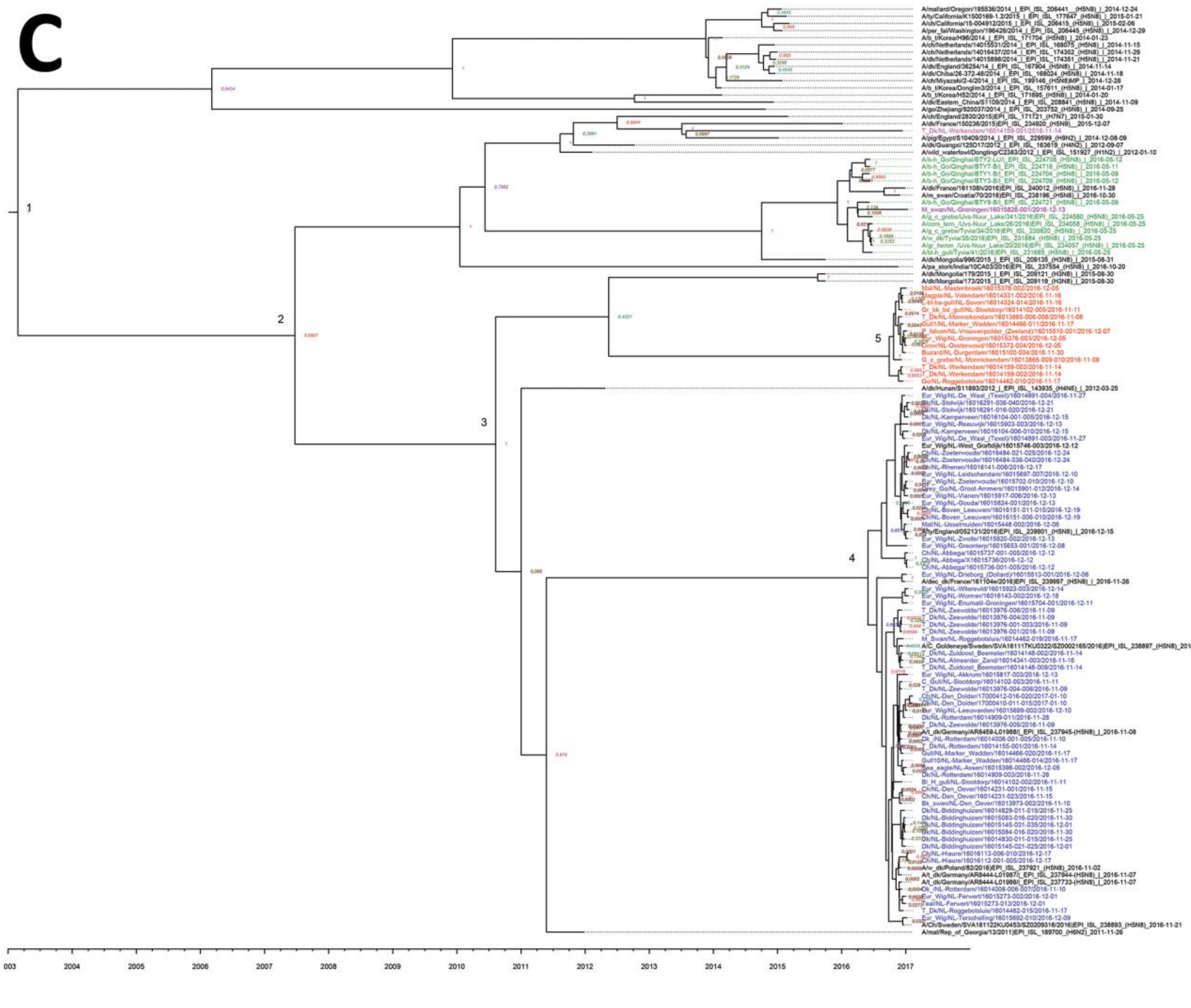
C
1
2
3
4
5
003
2004
2005
2006
2007
2008
2009
2010
2011
2012
2013
2014
2015
2016
2017

D

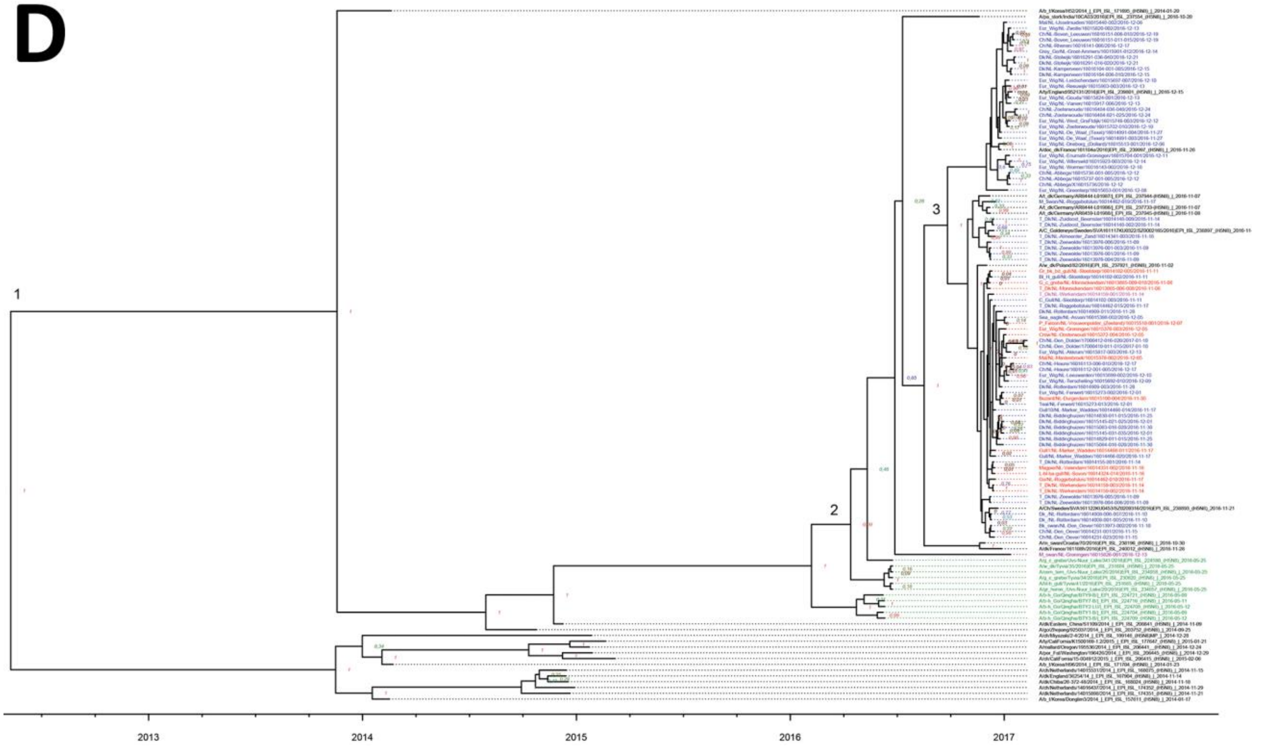

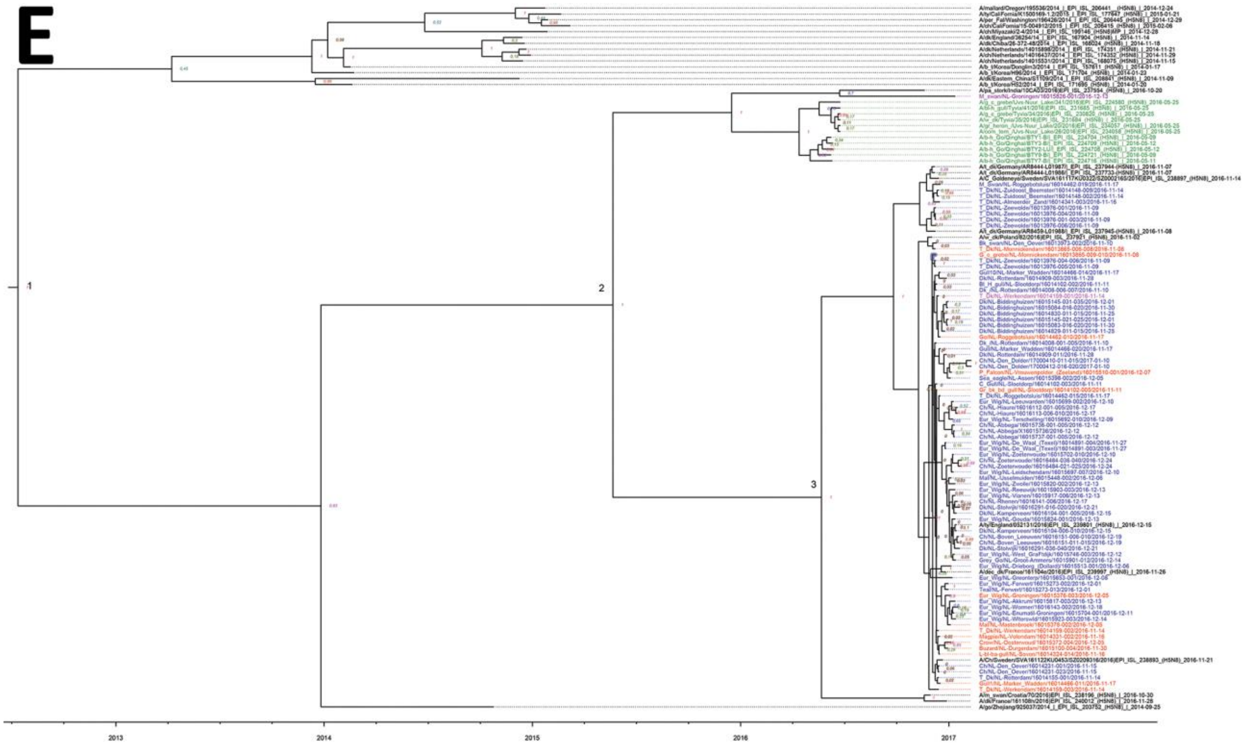
E
1
2
3
2013
2014
2015
2016
2017

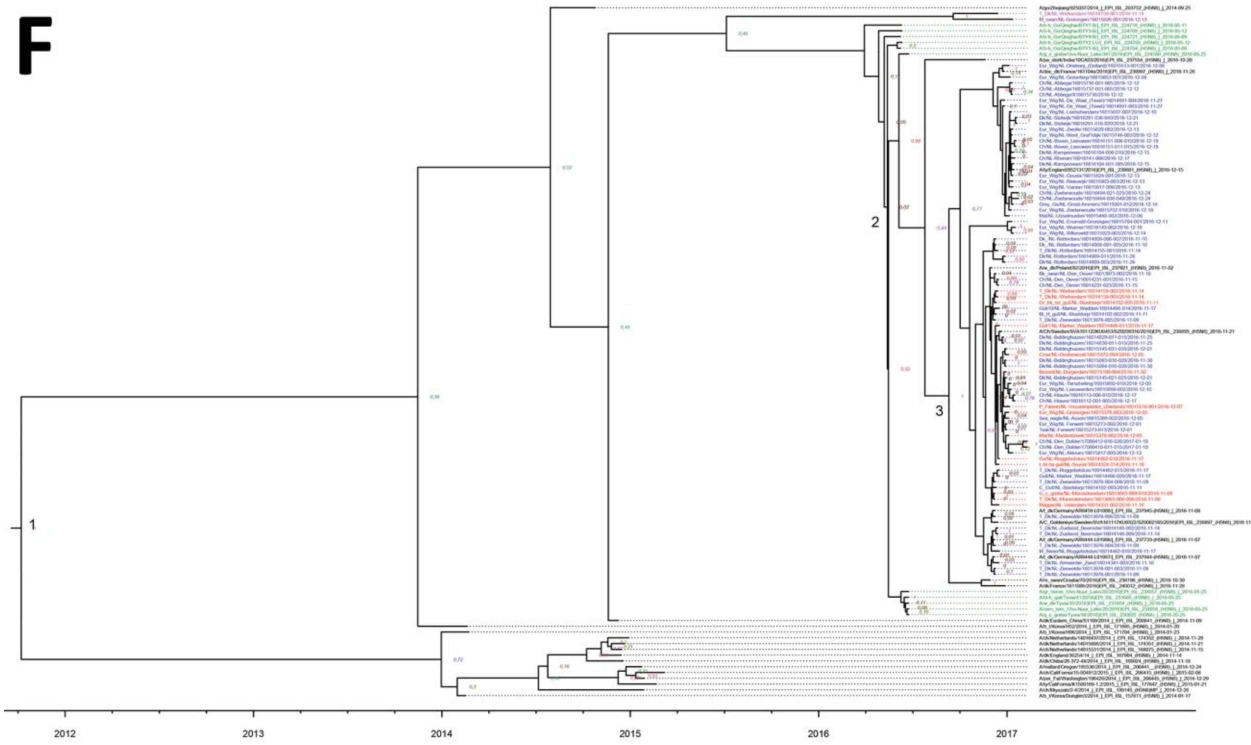
F
1
2
3
2012
2013
2014
2015
2016
2017

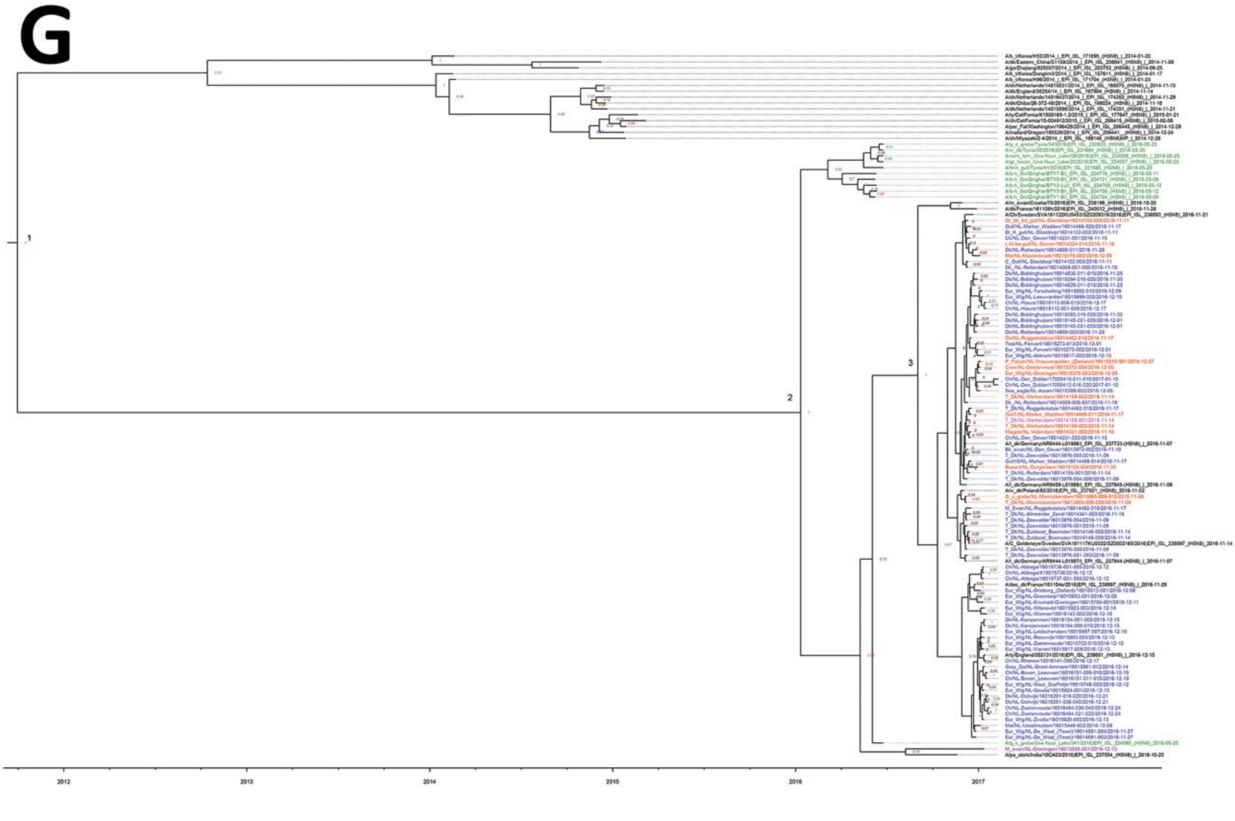
G
1
2
3
2012
2013
2014
2015
2016
2017

H

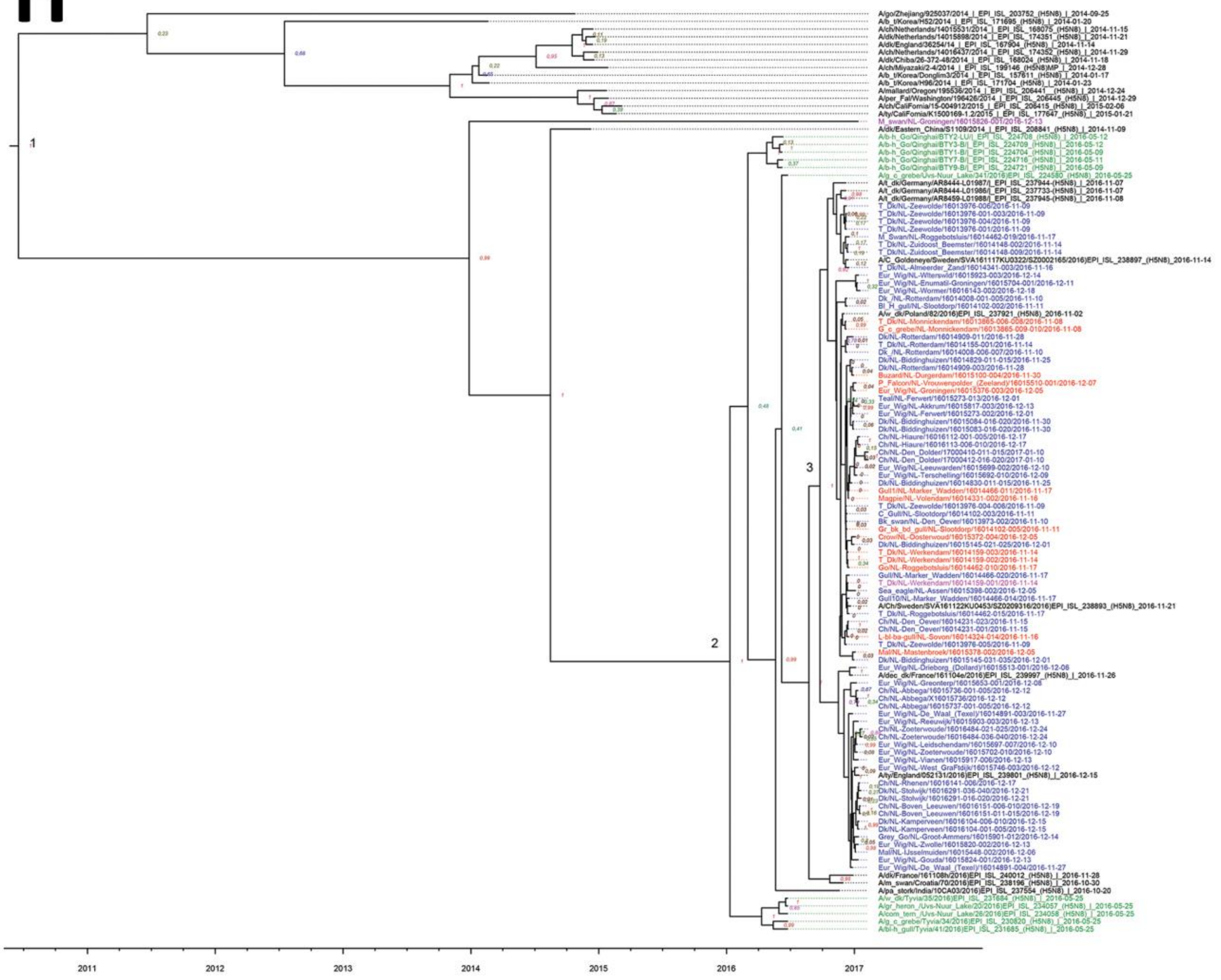

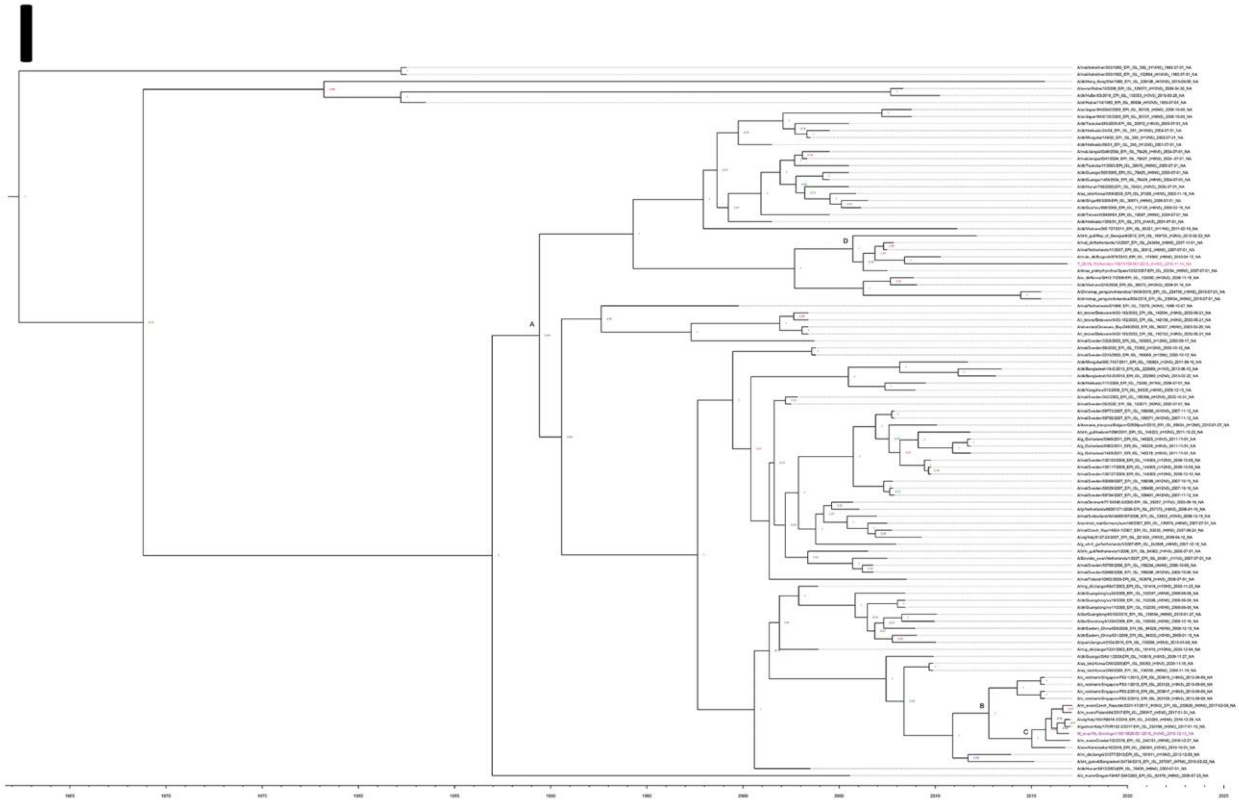

References

1. Saitou N, Nei M. The neighbor-joining method: a new method for reconstructing phylogenetic trees. Mol Biol Evol. 1987;4:406–25. PubMed

2. Tamura K, Nei M. Estimation of the number of nucleotide substitutions in the control region of mitochondrial DNA in humans and chimpanzees. Mol Biol Evol. 1993;10:512–26. PubMed

3. Tamura K, Stecher G, Peterson D, Filipski A, Kumar S. MEGA6: Molecular Evolutionary Genetics Analysis version 6.0. Mol Biol Evol. 2013;30:2725–9. PubMed http://dx.doi.org/10.1093/molbev/mst197
